# Loss of TDP-43 causes ectopic endothelial sprouting and migration defects through increased *fibronectin*, *vcam 1* and *integrin α4/β1*

**DOI:** 10.3389/fcell.2023.1169962

**Published:** 2023-06-13

**Authors:** Katrin Hipke, Bettina Pitter, Alexander Hruscha, Frauke van Bebber, Miha Modic, Vikas Bansal, Sebastian A. Lewandowski, Denise Orozco, Dieter Edbauer, Stefan Bonn, Christian Haass, Ulrich Pohl, Eloi Montanez, Bettina Schmid

**Affiliations:** ^1^ German Center for Neurodegenerative Diseases (DZNE), Munich, Germany; ^2^ Munich Cluster for Systems Neurology (SyNergy), Munich, Germany; ^3^ Walter Brendel Center, Biomedical Center, Ludwig-Maximilians-University Munich, Munich, Germany; ^4^ The Francis Crick Institute, London, United Kingdom; ^5^ Dementia Research Institute at KCL, London, United Kingdom; ^6^ National Institute of Chemistry, Ljubljana, Slovenia; ^7^ German Center for Neurodegenerative Diseases (DZNE), Tübingen, Germany; ^8^ Department of Medical Biochemistry and Biophysics (MBB), Karolinska Institute, Stockholm, Sweden; ^9^ Biomedical Center, Ludwig-Maximilians-University Munich, Munich, Germany; ^10^ Institute of Medical Systems Biology, Center for Biomedical AI (bAIome), Center for Molecular Neurobiology (ZMNH), University Medical Center Hamburg-Eppendorf, Hamburg, Germany; ^11^ DZHK (German Center for Cardiovascular Research), Partner Site Munich Heart Alliance, Munich, Germany; ^12^ Department of Physiological Sciences, Faculty of Medicine and Health Sciences, University of Barcelona and Bellvitge Biomedical Research Institute, Barcelona, Spain

**Keywords:** TDP-43, angiogenesis, neurodegeneration, zebrafish, ALS

## Abstract

Aggregation of the Tar DNA-binding protein of 43 kDa (TDP-43) is a pathological hallmark of amyotrophic lateral sclerosis and frontotemporal dementia and likely contributes to disease by loss of nuclear function. Analysis of TDP-43 function in knockout zebrafish identified an endothelial directional migration and hypersprouting phenotype during development prior lethality. In human umbilical vein cells (HUVEC) the loss of TDP-43 leads to hyperbranching. We identified elevated expression of *FIBRONECTIN 1* (*FN1*), the *VASCULAR CELL ADHESION MOLECULE 1* (*VCAM1*), as well as their receptor *INTEGRIN α4β1* (*ITGA4B1*) in HUVEC cells. Importantly, reducing the levels of *ITGA4, FN1*, and *VCAM1* homologues in the TDP-43 loss-of-function zebrafish rescues the angiogenic defects indicating the conservation of human and zebrafish TDP-43 function during angiogenesis. Our study identifies a novel pathway regulated by TDP-43 important for angiogenesis during development.

## Introduction

Aggregation of TDP-43 into stable inclusions accompanied by nuclear clearing of TDP-43 is the most prominent pathological characteristic of FTD and ALS (Neumann et al., 2006). TDP-43 delocalization and aggregation is thought to compromise TDP-43 function in the nucleus, ultimately leading to neurodegeneration. Addressing this hypothesis *in vivo*, loss-of-function animal models have been generated to investigate the physiological function of TDP-43 (Xu, 2012). Loss of TDP-43 is lethal in flies, zebrafish, and mice (Feiguin et al., 2009; Kraemer et al., 2010; Sephton et al., 2010; Wu et al., 2010; Schmid et al., 2013) challenging the identification of regulated pathways of TDP-43 *in vivo*. Conditional global knockout of TDP-43 in adult mice is lethal after 9 days of TDP-43 inactivation (Chiang et al., 2010) and cell-type specific TDP-43 knockout in motor neurons causes ALS-like motor neuron death (Wu et al., 2012; Iguchi et al., 2013), but the cause of death in these models remains unclear.

We previously generated a loss-of-function mutant for the human TDP-43 gene (*TARDBP*) encoding zebrafish orthologues, *tardbp* and *tardbp-like* (*tardbpl*) (Schmid et al., 2013). Homozygous loss of either *tardbp* or *tardbpl* does not cause any phenotype due to compensation by the other orthologous gene (Hewamadduma et al., 2013; Schmid et al., 2013). In contrast, the double homozygous mutants are affected by multiple defects, including reduced outgrowth of motor neurons, degeneration of muscle cells, as well as mis-patterning and non-perfusion of blood vessels, which together ultimately leads to early death at about 6–8 days post fertilization (dpf) (Schmid et al., 2013). The mutant vascular mis-patterning phenotype is very severe and is the first visible phenotype during development in the mutants. A growing body of evidence links vascular dysfunction to neurodegeneration (Zacchigna et al., 2008; Zlokovic, 2008), which prompted us to identify the molecular mechanism leading to the vascular phenotype in loss-of-function mutants of the ALS/FTLD gene *tardbp*. Given the large overlap in growth and guidance of the vasculature and neurons during development (Carmeliet and Tessier-Lavigne, 2005; Quaegebeur et al., 2011), these changes of pathways in the endothelium of TDP-43 mutants might be also essential in motor neurons and of relevance in ALS.

We therefore aimed at identifying the molecular pathways by which TDP-43 regulates angiogenic sprouting. First, we characterized a vascular mis-patterning phenotype upon loss of TDP-43 in zebrafish and human derived endothelial cells (ECs). Second, we identified de-regulated genes mediating the vascular TDP-43 deficient phenotype by conducting next-generation RNA sequencing (NGS) in TDP-43 knockdown (KD) HUVEC. Third, we rescue the zebrafish phenotypes by reducing the levels of Vcam1, Fn, and Itgα4 in TDP-43 knockout zebrafish. Lastly, we identified *FN1* as a direct RNA target of TDP-43 in HUVEC by performing iCLIP experiments.

## Materials and methods

### Zebrafish

Zebrafish embryos (*Danio rerio*) were kept at 28.5°C and were staged according to Kimmel et al. (1995). The wild type line AB was used for all experiments unless stated otherwise. All experiments were performed in accordance with animal protection standards of the DZNE and were approved by the government of Upper Bavaria (Regierung von Oberbayern, Munich, Germany).

The following mutant and transgenic alleles were used: *tardbp* ^*mde198−/−*^ (Schmid et al., 2013); *tardbp* ^*mde159−/−*^ (Schmid et al., 2013); *tardbpl* ^*mde222−/−*^ (Schmid et al., 2013); Tg (fli1:EGFP)^y1^ (Roman et al., 2002); Tg (fli1:nlsEGFP)^y7^ (Roman et al., 2002); Tg (kdrl:HRAS-mCherry)^s896^ (Chi et al., 2008).

### *In situ* hybridization

Whole mount *in situ* hybridization was performed as previously described (Schmid et al., 2013). The *in situ* probes used were previously described: *ephrin B2a* (Lawson et al., 2001), *flt4* (Lawson et al., 2001), *mflt1* (Bussmann et al., 2007), *plxnD1* (Torres-Vazquez et al., 2004), *sema3aa* (Yee et al., 1999), *sema3ab* (Torres-Vazquez et al., 2004). A 1 Kb fragment from the *sflt1* 3′ UTR has been amplified by PCR from embryonic cDNA (forward primer: sflt1 3’ UTR/reverse primer: flt1-UTR) and subcloned to generate the plasmid pCS2+GW-A + sflt1-UTR for antisense probe generation.

### Zebrafish immunohistochemistry

Embryos were fixed overnight (ON) at 4°C for 4 h at room temperature (RT). Next, they were rinsed 1x with PBS and washed three times for 5 min with PBST (PBS-Tween) at RT. For co-staining of 24 hpf Tg (fli1:EGFP)^y1^ embryos with antibodies specific for GFP and α-dystroglycan, embryos were treated with a methanol series of 30%, 60%, and 100% methanol in PBST for 5 min at RT. The samples were then stored ON or longer in 100% methanol at −20°C. Prior to the staining the embryos were rehydrated by a reverse methanol series of 60% and 30% methanol in PBST for 5 min at RT. Afterwards, the samples were washed three times for 5 min in PBST at RT. The embryos were blocked for 1 h in 10% NCST (Sigma, St. Louis, MO, United States). Incubation with primary antibodies was conducted at 4°C ON. On the next day, samples were rinsed with PBST and washed four times for 30 min in PBST at RT. Then they were blocked two times for 30 min in NCST. Secondary antibodies were used at a dilution of 1:500 in NCST and incubated at 4°C ON. The secondary antibodies were removed and the samples rinsed once with PBST followed by at least three washing steps for 15 min in PBST. The stained embryos were kept at 4°C in PBST or PBS until imaging with the spinning disk or confocal microscopes (Zeiss, Jena, Germany).

### Morpholino injections

All morpholinos (MO) were purchased from Gene Tools (*Philomath, OR,* United States)*.* Approximately 2 nL of MO were microinjected into fertilized zebrafish eggs at the 1 cell stage into the yolk.

**Table:** 

Name	Sequence	Concentration
*tardbp*-5′UTR	CAA​TAA​ACA​ACT​GCT​CGG​GTC​CAG​T	0.5 mM
*tardbp*-ATG	CTC​GAA​TGT​ACA​TCT​CGG​CCA​TCT​T	0.5 mM
*tardbp*-e3i3	TTT​TAC​CTG​CAC​CAT​GAT​GAC​TTC​C	0.5 mM
*tardbpl*-ATG	ATA​GCA​CTC​CGT​CAT​GAT​TAC​ACC​G	0.5 mM
*tardbpl*-e2i2	CTA​ACC​TGC​ACC​ATG​ATC​ACC​TCT​C	0.5 mM
*vcam1*-e1i1	CTA​ACA​GAT​GAA​ACT​TAC​CTG​CAA​C	0.75 mM
*itga4* -e2i2	GTA​ATG​GAG​GGA​AAA​CCT​ACC​AAC​A	0.5 mM
*itgaV* -ATG	CGG​ACG​AAG​TGT​TTG​CCC​ATG​TTT​T	1 mM
was previously described (Liu et al., 2012)		
*fn1*a mo1 [162]	TTT​TTT​CAC​AGG​TGC​GAT​TGA​ACA​C	0.75 mM
was previously described (Trinh and Stainier, 2004)		
*itga5*-5′UTR [165]	TAACCGATVTATCAAAATCCACTGC	0.5 mM
*fn1b* mo1 [165]	TAC​TGA​CTC​ACG​GGT​CAT​TTT​CAC​C	0.5 mM
*fn1b* mo2 [165]	GCT​TCT​GGC​TTT​GAC​TGT​ATT​TCG​G	0.5 mM

*itga5*-5′UTR and *fn1b* morpholinos were previously described (Julich et al., 2005).

Primer for KD validation of successful *itgα4* and *vcam1* KD.

KS A53 dr-itga4-Ex17-18 F AGG​TTT​CTG​CTC​GTT​TGG​TT
KS A54 dr-itga4-3UTR R CTT​TCA​TGC​TTG​GGC​ACA​TA
KS A55 dr-vcam1-ex1-2 F GCT​TTC​TTG​CTG​ACT​TTG​CT
KS A56 dr-vcam1-ex1-2 R GCA​TCT​CAG​CTC​ATT​CCT​GTC

### Cell transplantation

The transplantation experiments were conducted as previously described (Kemp et al., 2009). Coinjection of 3% dextran cascade blue (Molecular Probes, Eugene, OR, United States) was used to label donor derived cells. In 3 independent transplantation experiments a total of 96 donors and recipients survived. n = 4 wild type and n = 4 mutant donors with EC labeling transplanted in wild type recipients were scored.

### Antibodies

β-tubulin, T6199 (Sigma-Aldrich, St. Louis, MO, United States), WB: 1:10,000
AKT, 9272 (Cell Signaling, Danvers, MA, United States), WB: 1:1,000
Alexa Fluor 405 anti-mouse, A-31553 (Invitrogen, Carlsbad, CA, United States), IF 1:500
Alexa Fluor 405 anti-rabbit, A-31556 (Invitrogen, Carlsbad, CA, United States), IF 1:500
Alexa Fluor 488 anti-mouse, A-11029 (Invitrogen, Carlsbad, CA, United States), IF 1:500
Alexa Fluor 488 anti-rabbit, A-11034 (Invitrogen, Carlsbad, CA, United States), IF 1:500
Alexa Fluor 488-conjugated Isolectin-B4, I21411 (Life Technologies, Carlsbad, CA, United States), IF: 1:300
Alexa Fluor 568 anti-rabbit, A-11011 (Invitrogen, Carlsbad, CA, United States), IF 1:500
Alexa Fluor 622 anti-rabbit, A-21070 (Invitrogen, Carlsbad, CA, United States), IF 1:500
anti-rabbit-HRP, W401B (Promega, Madison, WI, United States), WB 1: 10,000
anti-mouse-HRP, W402B (Promega, Madison, WI, United States), WB 1:5,000
α-dystroglycan, NCL-b-DG (Leica Biosystems, Nußloch, Germany), IF: 1:50
Calnexin, SPA-860 (Stressgen, Victoria, BC, Canada), WB: 1:10,000
ERK1/2, 9102 (Cell Signaling, Danvers, MA, United States), WB: 1:2000
Fibronectin, HPA027066 (Sigma-Aldrich, St. Louis, MO, United States), WB: 1:500
GFP, 632375 (Clontech, Danvers, MA, United States), IF: 1:500
GFP, 632592 (Clontech, Danvers, MA, United States), IF: 1:500
Integrin α4, 8440 (Cell Signaling, Danvers, MA, United States), WB: 1:1,000
Integrin β1, 610467 (BD, Franklin Lakes, NJ, United States), WB: 1:2000
Tardbp, 4A12-111 (Helmholtz Center, Munich, Germany), WB: 1:1 (Schmid et al., 2013)
TDP-43 N-term, SAB4200006 (Sigma-Aldrich, St. Louis, MO, United States), WB: 1:10,000–1:50,000
p38, 9212 (Cell Signaling, Danvers, MA, United States), WB: 1:1,000
pAKT, 9271 (Cell Signaling, Danvers, MA, United States), WB: 1:500
pERK1/2, 9101 (Cell Signaling, Danvers, MA, United States), WB: 1:2000
PLCG1, sc-426 (Santa Cruz Biotechnology*, Dallas, TX,* United States), WB: 1:200
p-p38, 9211 (Cell Signaling, Danvers, MA, United States), WB: 1:1,000
pPLCG1, SAB4300082 (Sigma-Aldrich, St. Louis, MO, United States), WB: 1:500
pPI3K, 4228 (Cell Signaling, Danvers, MA, United States), WB: 1:1,000
pVEGFR2, 2478 (Cell Signaling, Danvers, MA, United States), WB: 1:1,000
VEGFR2, 9698 (Cell Signaling, Danvers, MA, United States), WB: 1:1,000

### Western blot analysis

Zebrafish embryos were lysed in 10–15 μL Laemmli buffer per embryo, homogenized, and heated at 95°C for 5 min. Lysates were centrifuged at 13,000 rpm and supernatant was loaded on a 12% SDS-PAGE gels (7% gels for VEGFR2 and PLCG1) and transferred to a PVDF membrane (Merck Millipore, Burlington, MA, United States) for 70 min at 400 mA. Membranes were blocked in 3% milk powder (Carl Roth, Karlsruhe, Germany), membranes for phosphorylated epitopes were blocked in 0.2% I-Block (Thermo Fisher, Waltham, MA, United States), and membranes for FN1 were blocked in 5% BSA (Sigma-Aldrich, St. Louis, MO, United States) for 60 min at RT. Incubation of primary antibody was performed ON at 4°C followed by 4 PBST washes and incubation in secondary antibody for 1 h at RT. After 6 washes with PBST membranes were developed with ECL Plus (Fisher Scientific, Waltham, MA, United States). The intensity of the bands was quantified using ImageJ. Each protein of interest was normalized to calnexin and significant changes between shCtr and sh*TARDBP* determined by paired *t*-test analysis (n = 3).

### shRNAS

**Table:** 

shCtr sense	gat​ccc​cCG​TAC​GCG​GAA​TAC​TTC​GAt​tca​aga​ga
	TCG​AAG​TAT​TCC​GCG​TAC​Gtt​ttt​gga​aa
shCtr antisense	ttt​cca​aaa​aCG​TAC​GCG​GAA​TAC​TTC​GAt​ctc​ttg​aa
	TCG​AAG​TAT​TCC​GCG​TAC​Ggg​gga​tc
sh*TARDBP* **#**1 sense	gat​ccc​cGG​AGA​GGA​CTT​GAT​CAT​TAt​tca​aga​ga
	TAA​TGA​TCA​AGT​CCT​CTC​Ctt​ttt​gga​aa
sh*TARDBP* **#**1 antisense	ttt​cca​aaa​aGG​AGA​GGA​CTT​GAT​CAT​TAt​ctc​ttg​aa
	TAA​TGA​TCA​AGT​CCT​CTC​Cgg​gga​tc
sh*TARDBP* **#**2 sense	gat​ccc​cGG​GTA​TGA​TGG​GCA​TGT​TAt​tca​aga​ga
	TAA​CAT​GCC​CAT​CAT​ACC​Ctt​ttt​gga​aa
sh*TARDBP* **#**2 antisense	ggg​CCC​ATA​CTA​CCC​GTA​CAA​Taa​gtt​ctc​t
	ATT​GTA​CGG​GTA​GTA​TGG​Gaa​aaa​cct​ttt​cga

### Chemical treatment of zebrafish

The treatment with chemical inhibitors was performed prior to outgrowth of ISA and it was hence not possible to differentiate *tardbp−/−; tardbpl−/−* embryos from their wild type siblings at this stage, *tardbp−/−;*Tardbpl KD and their control MO injected *tardbp −/−* siblings were used for testing the rescue potential of the applied chemical inhibitors. The VEGFR2 inhibitor DMH4 (Tocris Bioscience, Bristol, United Kingdom, 4,471) was applied at the 16- to 17-somite stage in different concentrations: 0.1, 0.5, and 2.5 μM. 1% DMSO served as a negative control. Embryos were kept till they reached the required developmental stage that was determined by status of ISA growth according to (Isogai et al., 2003). Next, they were fixed in 4% PFA ON at 4°C and imaged by confocal or spinning disk microscopes (Zeiss, Jena, Germany) at ×25 magnification.

### Reverse transcription and quantitative PCR

Total RNA was extracted using RNeasy Mini Kit (Qiagen, Hilden, Germany) or TRIzol (Thermo Fisher Scientific, Waltham, MA, United States) according to the manufacturer’s protocol and reverse transcribed with random hexanucleotide primers using the M-MLV Reverse Transcription kit (Invitrogen) with RiboLock RNase inhibitor (Thermo Fisher Scientific, Waltham, MA, United States). The complementary DNA was amplified using qPCR SsoFast Evagreen Supermix (Bio-Rad, Hercules, CA, United States) and analyzed in the C1000 Thermal Cycler (Bio-Rad). Each reaction was conducted as technical triplicates with at least 3 biological replicates. The relative expression of each gene was calculated using the ΔΔCT-method and the normalized fold expression was calculated by normalization to the reference genes *rpl13a* and *elf1a2*.

The following primers were used for qPCR.

**Table:** 

oA03 β-actin F	TGT​TTT​CCC​CTC​CAT​TGT​TGG
oA04 β -actin R	TTC​TCC​TTG​ATG​TCA​CGG​AC
KS A1 sema3ab_I1_F	AAC​GTA​CCC​CGG​CTT​AAA​CT
KS A2 sema3ab_I1_R	GCA​GAG​CTG​TTA​GCC​AAT​CC
KS A3 sema3aa_I1_R	TCC​ATC​AGG​AAC​GTG​TCG​TA
KS A4 sema3aa_I3_F	CTT​CCA​AAC​GCG​ATG​AAT​G
KS A5 Vegfr2_I11_F	TCC​TCT​TCC​CAT​TGA​AAA​CG
KS A6 Vegfr2_I11_R	CTG​TTT​TCA​CCA​CCA​GGG​TA
KS A7 mflt_I24_F	TGG​TCA​TAT​GGA​GTC​CTG​CTC
KS A8 mflt_I24_R	AGG​AGA​ACA​CAT​CCG​AGT​GC
KS A9 sflt_E10-11c_F	GTC​CCA​CCA​CCT​CAA​ATC​C
KS A10 sflt_E10-11c_R	GGC​CCA​CAA​CTC​CAC​TCT​C
KS A11 Rpl13a_E3-4a_F	ATT​GTG​GTG​GTG​AGG​TGT​GA
KS A12 Rpl13a_E3-4a_R	CAT​TCT​CTT​GCG​GAG​GAA​AG
KS A13 elf1a2 F	AGC​AGC​AGC​TGA​GGA​GTG​AT
KS A14 elf1a2 R	GTG​GTG​GAC​TTT​CCG​GAG​T
KS A43 dr-vcam1 ex9-10 F	CAA​ACG​ACC​TGG​GTT​ACG​AA
KS A44 dr-vcam1-ex9-10 R	CAG​CAG​AAC​CTC​CCA​AGA​AA
KS A45 dr-itga4-ex2-3 F	TGC​AGT​ATG​TTG​AAC​AGC​CAG
KS A46 dr-itga4-ex2-3 R	CAA​ACT​CAC​ACC​CAG​CCA​C
KS A47 dr-fn1a-ex3-4 F	TGT​ACT​TGC​ATT​GGC​TCT​GC
KS A48 dr-fn1a-ex3-4 R	GTC​TCT​GCC​ATG​TGT​CTC​CA
KS A49 dr-fn1b-ex39-40 F	CAT​TGC​CCT​TCT​GAA​TAA​CCA
KS A50 dr-fn1b-ex39-40 R	ATG​ACT​GGG​CAG​GCT​AGG​TA

### HUVEC

HUVEC were purchased from PromoCell (Heidelberg, Germany) and cultured in 50% Endothelial Cell Basal Medium (PromoCell, Heidelberg, Germany) and 50% Medium 199 (Life Technologies, Carlsbad, CA, United States) with 20% FCS. VEGF-A stimulation was performed 5 days post transduction with 25 ng/μL VEGF-A for 0, 5, 15, or 30 min at 37°C and 5% CO_2_ respectively. The cells were subsequently washed twice with ice cold PBS and further processed with RIPA with 1x phosphatase and protease inhibitor for SDS-PAGE or TRIZOL for RNA analysis.

For virus transduction, cells were grown to 80% confluence and detached with Trypsin/EDTA. They were seeded and transduced with virus containing either the sequence coding for shCtr, sh*TARDBP* **#**1, or sh*TARDBP* **#**2 on the following day with 25 μL virus mix (described below) per 10 mL culture volume in a 10 cm dish.

The Tube formation assay was performed on the artificial extracellular matrix Geltrex (Thermo Fisher Scientific, Waltham, MA, United States) according to the manufacturer’s protocol “Endothelial Tube formation assay (*in vitro* Angiogenesis)”.

### Virus production

For virus production, HEK 293-FT cells were used as packaging cells at low passage number and only grown to 60%–70% confluence in 10 cm dishes. One day after the last passaging, cells were transfected with three different constructs, two that code for the lentiviral particles (pVSVg and pSPAX) and with the construct of interest. Before transfection, medium was replaced by 5 mL pre-warmed Optimem (Invitrogen, Carlsbad, CA, United States) with 10% FCS. Then, per three 10 cm dishes to be transfected, 108 μL Lipofectamin 2000 (Thermo Fisher Scientific, Waltham, MA, United States) was mixed with 4,500 μL Optimem (L2K) and incubated for 5 min. The transfection mix contains 18.6 μg of the construct of interest, 11.0 μg pSPAX2, and 6.4 μg pVSVg with 4,500 μL Optimem. This mix was combined with the L2K and incubated for 20 min at RT. Finally, 3 mL per mix was added to one 10 cm dish. Next day, media were replaced by 10 mL high packaging media (1% penicillin/streptomycin, 1% NEAA and 10% FCS in DMEM Glutamax (all from Thermo Fisher Scientific, Waltham, MA, United States). On the second day after transfection, the supernatant of three 10 cm dishes containing virus with the same sequence of interest was pooled in 50 mL tubes and centrifuged for 5 min at 2000 rpm to remove debris. The supernatant was filtered through a 0.45 μm PES membrane filter (Whatman, Little Chalfont, Buckinghamshire, United Kingdom) into 28SW ultracentrifuge tubes. The virus particles were ultracentrifuged for 2.5 h at 22,000 rpm. Supernatant was discarded and the virus particles resuspended over night at −4°C in 120 μL NB medium (Thermo Fisher Scientific, Waltham, MA, United States).

### RNA-sequencing analysis

RNA-sequencing resulted in ∼25–31 million reads (Supplementary Table S1). Quality assessment was based on the raw reads using the FASTQC quality control tool (v0.10.1). The sequence reads (single-end 50 bp) were aligned to the human reference genome (hg38) with Bowtie2 (v2.0.2) using RSEM (v1.2.29) with default parameters. First, the human reference genome was indexed using the Ensembl annotations (v84.38) with *rsem-prepare-reference* from RSEM software. Next, *rsem-calculate-expression* was used to align the reads and quantify the gene abundance. Differential expression analysis was carried out using gene read counts with DESeq2 package (v1.12.4). Genes with less than 5 reads (baseMean) were filtered out. Genes with an adjusted *p*-value ≤0.01 and log2 fold change > |0.5| were considered to be differentially expressed.

### Gene ontology enrichment analysis

Gene ontology (GO) analysis was conducted using WebGestalt. An adjusted *p*-value ≤0.01 using the Benjamini–Hochberg method for controlling the false discovery rate was set as significant for GO terms in biological processes. EnrichmentMap (v2.1.0) plugin was used in Cytoscape (v3.4.0) for enrichment visualization of top 20 terms (Supplementary Figure S5). The overlap between the gene sets (biological process) was calculated using overlap coefficient implemented in the plugin, i.e, [size of (A intersect B)]/[size of (minimum (A, B))].

### ICLIP analysis

The iCLIP protocol was performed as described previously (Tollervey et al., 2011), with the following modifications. Early passage HUVECs were irradiated once with 155 mJ/cm^2^ in a Stratlinker 2,400 at 254 nm. TDP-43 was immunoprecipitated with protein A Dynabeads (Invitrogen, Carlsbad, CA, United States) conjugated to rabbit-anti TDP-43 (Sigma, St. Louis, MO, United States, SAB4200006). The region corresponding to 55–100 kDa complexes was excised from the membrane to isolate the RNA. High-throughput sequencing using Illumina HiSeq was done. Analysis of reproducibility of crosslink sites, identification of the significant iCLIP crosslink clusters and z-score analysis of enriched pentamers was done as described previously (Tollervey et al., 2011) and data was processed by iCount webserver (http://icount.biolab.si).

## Results

### TDP-43 loss-of-function causes increased and ectopic angiogenic sprouting and impaired directional migration of endothelial cells (ECs) in zebrafish

**FIGURE 1:**
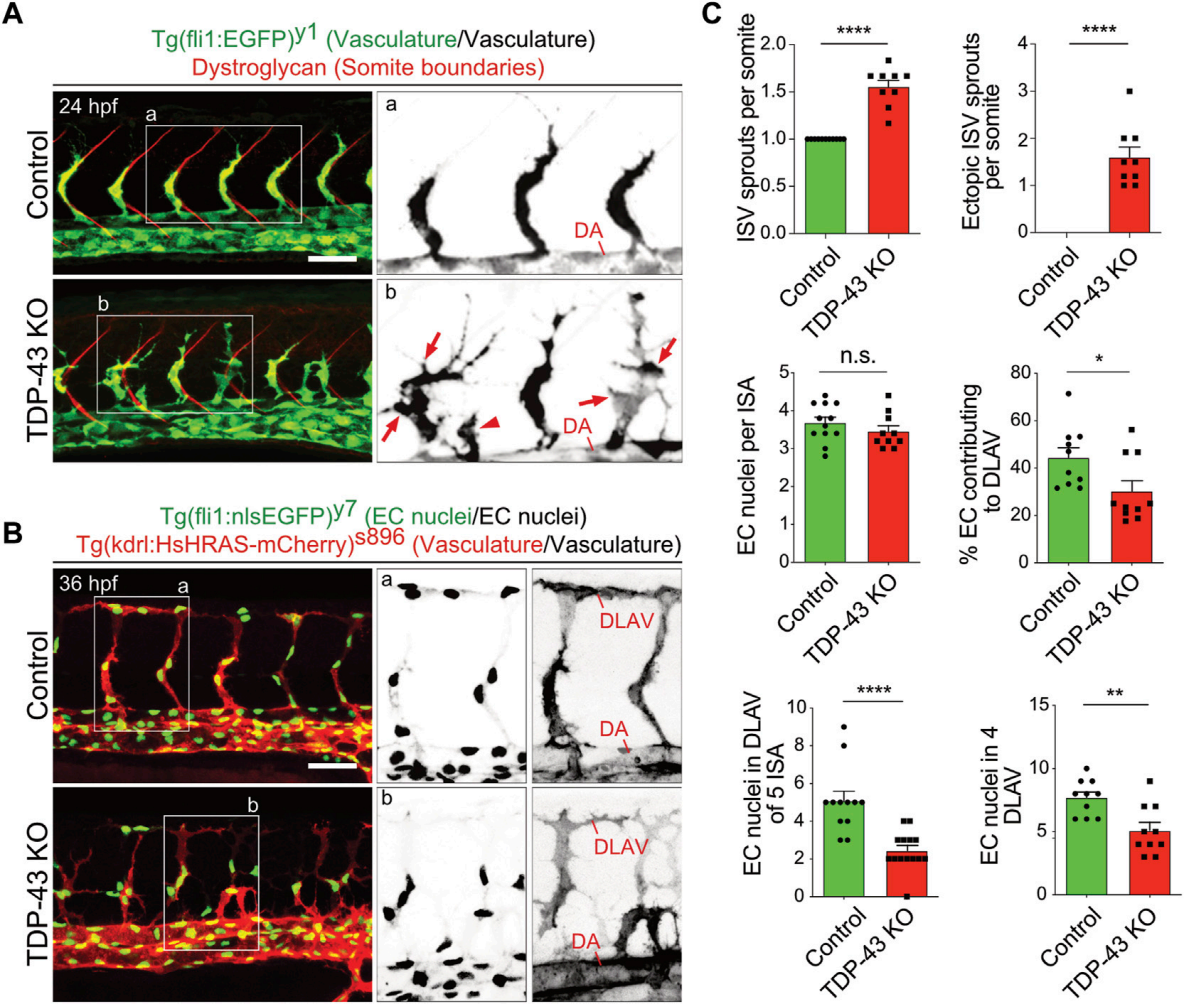
Loss of TDP-43 leads to increased vascular sprouting and defective migration. **(A)** More and ectopic SA sprouts in zebrafish *tardbp−/−; tardbpl−/−* mutants. Immunofluorescence of a sibling and a *tardbp−/−; tardbpl−/−* mutant embryo at 24 hpf. The Tg (fli1a:EGFP)^**y**1^ highlights the vasculature in green, the somite boundaries are stained in red. Scale bar 50 μm, anterior to the left. The insets a and b highlight the vasculature only. DA = dorsal aorta **(B)** Impaired directed migration in *tardbp−/−; tardbpl−/−* mutants. Less mutant EC nuclei have reached the DLAV at 32 hpf. Embryos are transgenic for Tg (fli1:nlsEGFP)^**y**7^ with nuclear EGFP expression (green) and Tg (kdrl:HsHRAS-mCherry)^**s**896^, highlighting the vasculature (red). Scale bar 50 μm, anterior to the left. The insets a and b highlight the EC nuclei (left) and the vasculature (right). **(C)** Quantifications of the vascular defects in the *tardbp−/−; tardbpl−/−* mutants as indicated. Kruskal–Wallis test and Dunn’s multiple comparison post test, n ≥ 9, sprouts between boundaries of six somites dorsal to the end of the yolk sack extension were quantified. D’Agostino-Pearson omnibus normality test and unpaired *t*-test, n = 10 for "% EC contributing to DLAV” and “number of EC in DLAV of 4 segments at 2.5 dpf”.

The initial characterization of *tardbp−/−; tardbpl−/−* double homozygous loss-of-function zebrafish mutants uncovered hyperbranching and mis-patterning of EC during development (Schmid et al., 2013). In zebrafish, the intersegmental arteries (SA) grow dorsally from the dorsal aorta (DA) in the zebrafish trunk at 24 h post fertilization (hpf) in a very stereotypic manner (Isogai et al., 2001; Hogan and Schulte-Merker, 2017). In wild type embryos only one sprout per somite is formed at every somite boundary. The main lamellipodium of the sprouting SA is directed dorsally towards the physiological target region of the growing sprout (Figure 1A). In contrast, in the *tardbp−/−; tardbpl−/−* mutants, more sprouts are formed also at ectopic positions between somite boundaries. Moreover, growing sprouts extend lateral lamellipodia that frequently connect with neighboring sprouts (Figures 1A, B). The direction of lamellipodia extension defines the directionality of cell migration (Petrie et al., 2009). The extension of lateral lamellipodia and migration of EC at ectopic positions along the DA hence indicates a defect in directed migration. In wild type embryos, directed migration of EC to the dorsal roof of the neural tube is followed by formation of lateral connections between EC of neighboring SA to form the dorsal longitudinal anastomotic vessel (DLAV) at 32 hpf (Isogai et al., 2001). To provide further evidence for impaired directed migration of EC in *tardbp−/−; tardbpl−/−* mutants, we quantified EC nuclei contributing to the DLAV at 32 hpf. EC nuclei in the DLAV of the analyzed 5 somites are drastically reduced in *tardbp−/−; tardbpl−/−* mutants (Figures 1B, C). We further excluded a delay in cell migration, since EC nuclei contributing to the DLAV in the analyzed 4 somites are still significantly decreased at 2.5 dpf (Figure 1C). EC nuclei number per SA is not different in *tardbp−/−; tardbpl−/−* mutants compared to wild type siblings in line with impaired migration of EC during angiogenesis in *tardbp−/−; tardbpl−/−* mutants.

We next established a TDP-43 KD model for the subsequent experiments to obtain higher numbers of phenotypic embryos compared to the 25% of double homozygous mutant embryos obtained from a *tardbp−/−; tardbpl+/-* incross. The KD specificity was carefully evaluated by comparison to the *tardbp−/−; tardbpl−/−* mutant phenotypes. The KD of Tardbpl protein in the *tardbp−/−* background (*tardbp−/−;* Tardbpl KD) can fully phenocopy the *tardbp−/−; tardbpl−/−* mutant EC phenotype demonstrating the specificity and efficiency of the morpholino (MO) (Supplementary Figures S1A–C).

### TDP-43 deficient endothelial phenotypes are not mediated by Vegfr and Notch signaling

**FIGURE 2:**
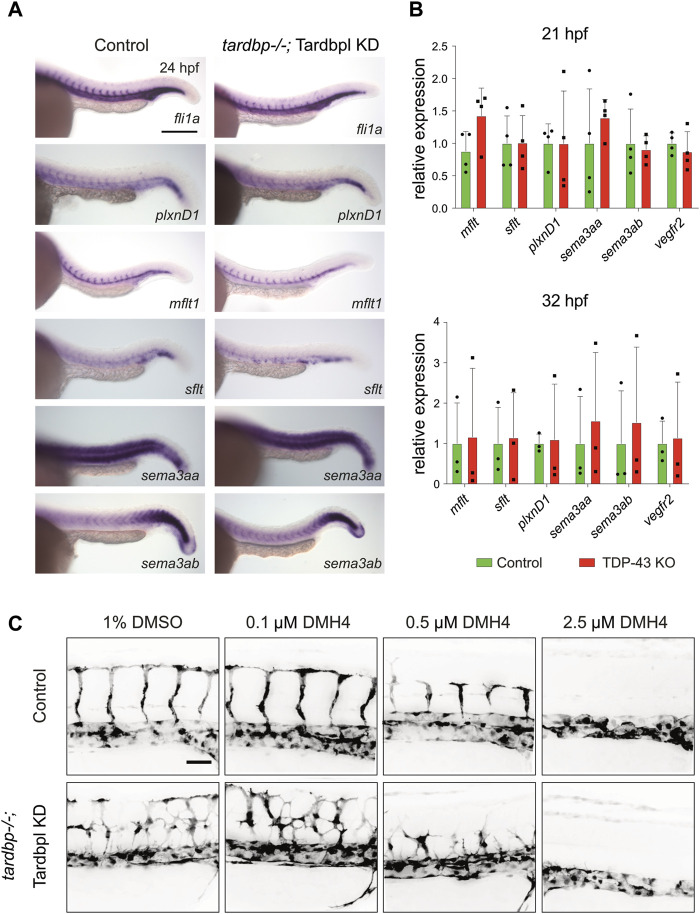
Selected candidate genes and the VEGF pathway are not affected in TDP-43 KO zebrafish **(A)** WISH with antisense probes specific for the candidate genes *fli1a, plxnD1, sflt1, mflt1, sema3aa, and sema3ab* in *tardbp−/−* ctr MO injected embryos (Control) and their *tardbp−/−;* Tardbpl KD siblings at 24 hpf (Magnification: ×10 anterior to the left). Depicted are representative images of embryos from one clutch. Experiment was performed with embryos of three independent clutches. **(B)** Relative mRNA expression of *mflt1, sflt1, plxnD1, sema3aa, sema3ab,* and *kdrl* in *tardbp−/−;* Tardbpl KD embryos compared to their ctr MO injected *tardbp−/−* siblings (Control) at 21 and 32 hpf. N = 3 (32 hpf) or n = 4 (21 hpf) pools of embryos of independent clutches, unpaired *t*-test, all *p*-values >0,5. Results were reproduced three times using the same cDNA. **(C)** Representative images of ctr MO injected siblings (Control) and *tardbp−/−*; Tardbpl KD embryos treated with 1% DMSO (solvent) as control or 0.1 μM, 0.5 μM, and 2.5 μM DMH4 inhibitor. Scale bar 50 μm, anterior to the left.

Numerous guidance cues and growth factors are known to regulate angiogenic sprouting of SA in the zebrafish embryo. The similarity of the PlxnD1 loss-of-function mutant *out of bounds (obd)* to the *tardbp−/−; tardbpl−/−* mutant phenotype indicated a possible impairment of this signaling pathway in *tardbp−/−; tardbpl−/−* EC (Childs et al., 2002; Torres-Vazquez et al., 2004; Zygmunt et al., 2011). The zebrafish homologue of VEGFR1, *flt1* (fms-related tyrosine kinase)*,* is alternatively spliced into a soluble decoy receptor, *sflt1*, and a membrane bound version, *mflt1*. In *obd* mutants, the ratio of the soluble *sflt1* and membrane bound *mflt1* is shifted towards increased expression of the *mflt1* splice variant, leading to an increased availability of Vegf for its pro-angiogenic receptor Kdrl (Zygmunt et al., 2011), the zebrafish orthologue of VEGFR2. However, no alterations in expression patterns of *sflt1*, *mflt1*, *plxnD1* and its receptor *sema3aa* and *sema3ab* were detectable in TDP-43 deficient embryos by *in situ* hybridization (Figure 2A). The expression levels of *sflt1*, *mflt1*, *plxnD1*, *sema3aa*, *sema3ab*, and *kdrl*, were also not altered as determined by RT-PCR (Figure 2B). Thus, although phenotypically similar, the *tardbp−/−; tardbpl−/−* phenotype is not caused by the same alteration of guidance cues upstream of Kdrl signaling as in *obd* mutants.

Notch is another important regulator of angiogenic sprouting and could thus contribute to increased sprouting of SA in *tardbp−/−; tardbpl−/−* mutants. Besides the different location and timing of additional sprouting in Notch deficient embryos and *tardbp−/−; tardbpl−/−* mutants, the increased EC number in sprouting SA in embryos with Notch deficiency is not present in *tardbp−/−; tardbpl−/−* mutants (Figure 1C and (Siekmann and Lawson, 2007)). Further, Notch is an important player in EC fate determination. However, the Notch determined cell fate markers *ephrin-B2a* and *flt4* are not altered in *tardbp−/−;* Tardbpl KD embryos (Supplementary Figure S2), demonstrating that arterial-venous cell differentiation is normal. Taken together, there is no evidence for Notch-related phenotypes in *tardbp−/−; tardbpl−/−* mutants based on EC numbers and expression of marker genes.

VEGFR2 signaling influences a multitude of EC behaviors and vascular patterning (Simons et al., 2016) and is the mediator of various signaling molecules. Increased VEGFR2 signaling stimulates angiogenesis and could potentially explain excessive angiogenic sprouting as seen in *tardbp−/−; tardbpl−/−* mutants. If increased Kdrl signaling is indeed responsible for the sprouting phenotype, then slight reduction of Kdrl signaling with low concentrations of VEGFR2 inhibitor should be able to rescue the phenotype. To test this hypothesis, the selective VEGFR2 inhibitor DMH4 was used to slightly reduce Kdrl signaling to intermediate levels and assayed for rescue of the mutant phenotype (Cannon et al., 2010; Hao et al., 2010; van Rooijen et al., 2010). DMH4 was carefully titrated until sprouting was strongly impaired or completely absent in control (ctr) MO injected siblings. Then, the inhibitory effects of the intermediate concentrations (0.1 and 0.5 μM DMH4) that do not fully block sprouting were compared between TDP-43 deficient embryos and their ctr MO injected morphologically wild type siblings (Figure 2C). Even if sprouting is impaired in *tardbp−/−;* Tardbpl KD embryos at these intermediate concentrations, EC still show increased and ectopic sprouting indicating that Kdrl signaling is impaired but the hypersprouting phenotype is not rescued. Furthermore, titration of the DMH4 inhibitor to a 2.5 μM concentration that is just sufficient to block SA sprouting in *tardbp−/−* ctr MO siblings is equally sufficient to block angiogenic sprouting in *tardbp−/−;* Tardbpl KD embryos (Figure 2C) suggesting that Kdrl signaling is not increased in *tardbp−/−;* Tardbpl KD embryos.

### The TDP-43 KO vascular phenotype persists in a wild type environment

**FIGURE 3:**
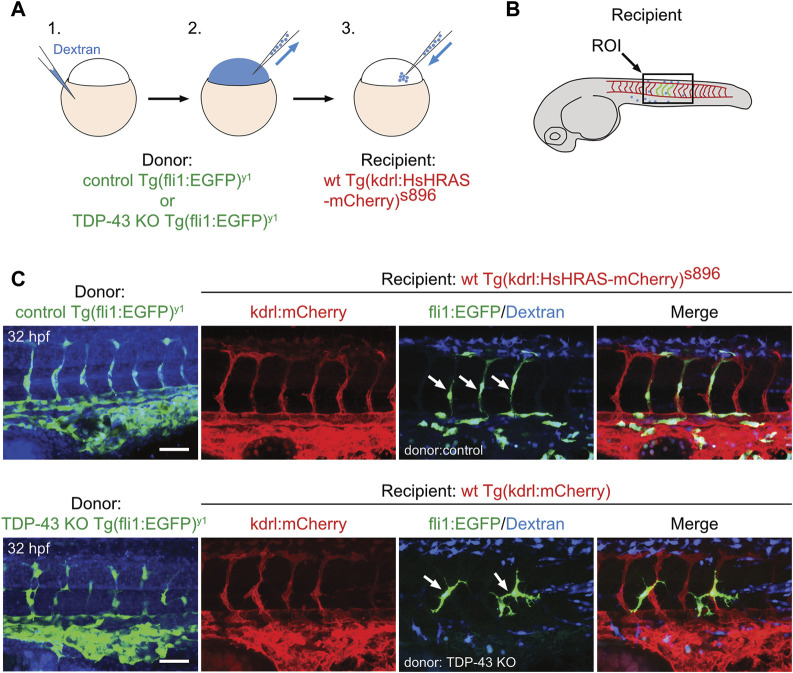
The *tardbp−/−; tardbpl−/−* mutant vascular phenotype is cell autonomous. **(A)** Scheme of the transplantation of control Tg (fli1:EGFP)^y1^ or TDP-43 KO; Tg (fli1:EGFP)^y1^ donor EC cells (EC in green, injected tracer label in blue) into Tg (kdrl:HsHRAS-mCherry)^s896^ hosts (EC in red). **(B)** The region of interest (ROI) imaged in the transplanted donor larvae is the area dorsal to the end of the yolk sack extension for all images (box). **(C)** Tg (fli1:EGFP)^y1^ donor (EC in green, injected tracer label in blue) transplanted into Tg (kdrl:HsHRAS-mCherry)^s896^ recipients (EC in red) had a wild type morphology. TDP-43 KO; Tg (fli1:EGFP)^y1^ mutant donor into Tg (kdrl:HsHRAS-mCherry)^s896^ recipient showed the TDP-43 deficient phenotype (n = 4). Scale bar 50 μm, anterior to the left. Displayed is the area dorsal to the end of the yolk sack extension for all images.

To test if a wildtype environment can rescue the mutant EC phenotype, we performed cell transplantation experiments of mutant EC into a wild type recipient embryo. Transplantation of donor cells from Tg (fli1:EGFP)^y1^ expressing eGFP in Tg (kdrl:mCherry)^s896^ recipients expressing mCherry in the endothelium does neither alter donor derived nor recipient EC morphology, showing that the transplantation procedure *per se* does not affect EC morphology (Figures 3A–C upper panel). When *tardbp−/−;* Tardbpl KD Tg (fli:GFP)^y1^ or *tardbp−/−; tardbpl−/−* Tg (fli:GFP)^y1^ embryos are used as donors, the transplanted cells differentiating into EC within the Tg (kdrl:mCherry^)s896^ recipient embryos show the mutant phenotype with supernumerous sprouts at ectopic positions (Figure 3C lower panel) consistent with a cell autonomous function of TDP-43. All donor cells were labeled with fluorescent dextran and only EC without co-transplanted dextran positive cells in the vicinity were scored.

### TDP-43 regulates branching of human ECs *in vitro* independent of VEGFR2 activation

**FIGURE 4:**
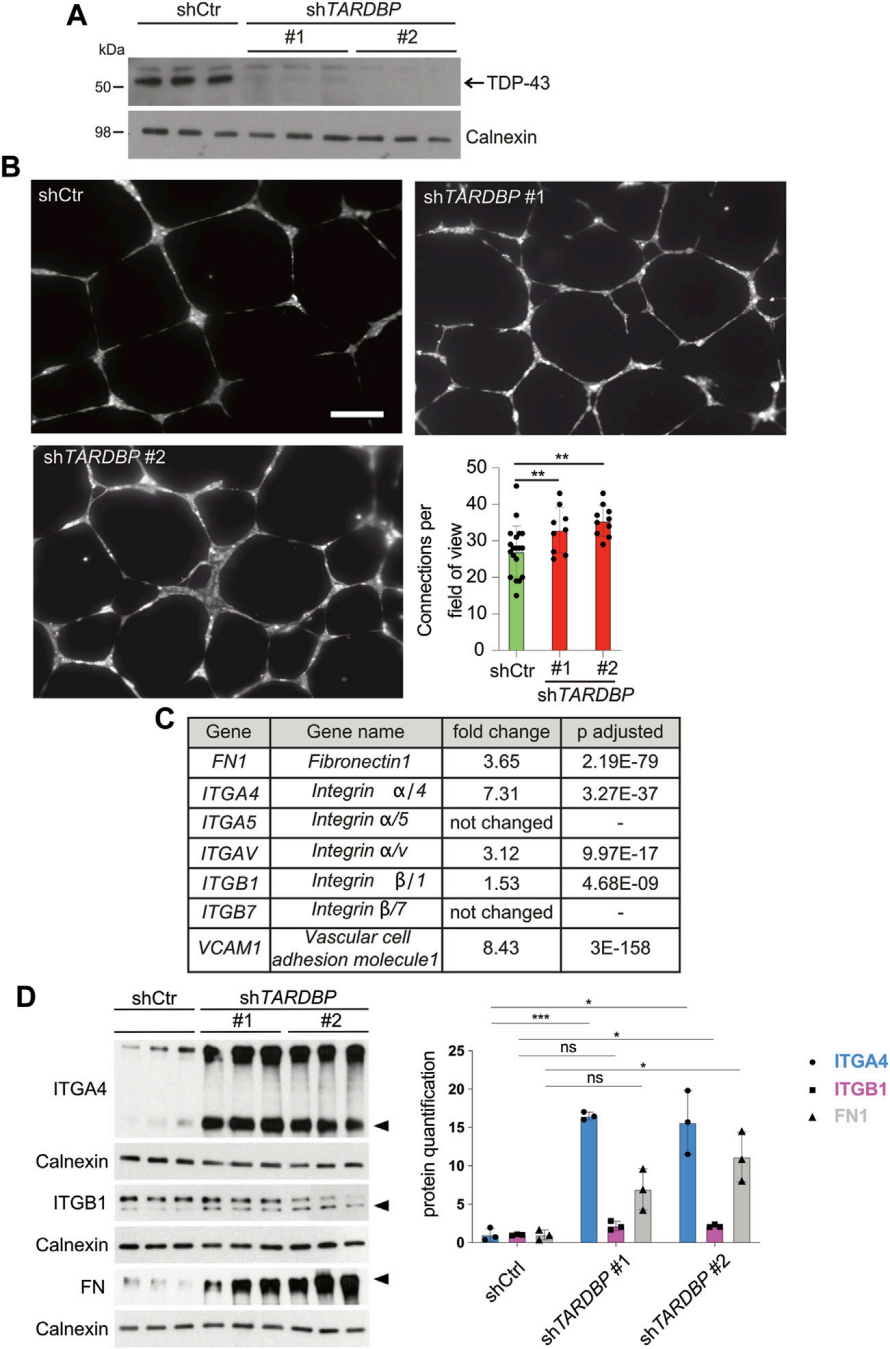
Increased *in vitro* angiogenesis upon TDP-43 KD in HUVEC and identification of mis-regulated pathways by NGS. **(A)** KD of TDP-43 in HUVEC with two different shRNAs, sh*TARDBP* **#**1 and **#**2 reduces TDP-43 protein levels. shCtr transduced HUVEC serve as control, each lane represents a biological replicate, Calnexin serves as loading control. **(B)** Images show tubular network formation of representative fields of view of shCtr, sh*TARDBP* **#**1, and sh*TARDBP* **#**2 transduced HUVEC (magnification: ×10). Bar graph displays the quantified connections per field of view in TDP-43 KD with sh*TARDBP*#1 and sh*TARDBP*#2 compared to shCtr. D’Agostino-Pearson omnibus normality test and one-way ANOVA, n ≥ 8 in three independent experiments. **(C)** Selection of NGS hits of HUVEC upon TDP-43 KD with fold change and adjusted *p*-value. **(D)** Immunoblot validation of FN1, ITGA4, and ITGB1 upon KD of TDP-43 with sh*TARDBP* **#**1 and sh*TARDBP* **#**2 in comparison to shCtr transduced cells. Each lane represents one biological replicate. The band intensities of the indicated bands (arrowhead) have been quantified using ImageJ, *t*-test n = 3.

We reasoned that the signaling pathways mediating the angiogenic phenotype are conserved in the human system and that identification of the molecular pathway is facilitated in a primary cell culture model. We used two independent *TARDBP* targeting shRNAs, sh*TARDBP*#1 and #2 (Figure 4A) to KD TDP-43 in HUVEC. A tube formation assay was performed to analyze collective migration on an artificial extracellular matrix (ECM) (Friedl and Gilmour, 2009), which is routinely used as an *in vitro* tube formation assay (Montañez et al., 2002; Friedl and Gilmour, 2009). Strikingly, network complexity (as reflected by the number of connections between branch points) is increased upon TDP-43 KD with both *TARDBP* targeting shRNAs (Figure 4B). This phenotype is not mediated by hyperproliferation, since TDP-43 KD does not affect proliferation, but rather leads to increased cell death (Supplementary Figure S3A). Thus, the TDP-43 loss-of-function affects branching and tube formation in zebrafish and human, suggesting an evolutionary conserved requirement of TDP-43 for EC patterning.

Next, we analyzed VEGFR2 signaling pathway activity and its responsiveness to a VEGF-A stimulus upon TDP-43 deficiency in HUVEC. Baseline activation after deprivation of growth factors was assessed as well as the time dependent increase in phosphorylation, and therefore kinase activity, upon VEGF stimulation. However, neither expression levels of the kinases nor the activation of VEGFR2 and the downstream kinases AKT, p38, PLCG and ERK1/2 were altered upon TDP-43 KD at any time point investigated (Supplementary Figure S3B and Supplementary Figure S4B). Thus, VEGFR2 signaling and responsiveness to VEGF, as well as kinetics of activation of kinases following a VEGF stimulus are not altered upon TDP-43 KD. Taken together, our data exclude involvement of impaired VEGFR2 signaling in the vascular phenotype observed in both, *tardbp−/−; tardbpl−/−* zebrafish and TDP-43 loss-of-function HUVEC.

### TDP-43 KD activates the FN1/VCAM1/ITGA4B1 pathway in HUVECs

After excluding prominent candidate pathways mediating the EC phenotype in TDP-43 KO we turned to RNA next-generation sequencing (NGS) as an unbiased approach to identify differentially expressed genes upon KD of TDP-43 in HUVECs. We identified 2,129 genes with differential expression and adjusted *p*-value smaller or equal to 0.01 and a minimum coverage of 5 reads (see Supplementary Table S1). Pathway analysis of the differentially expressed genes identifies migration, extracellular structure organization, chemotaxis and morphogenesis defects as the most prominent hits (Supplementary Figure S5 and Supplementary Table S2). Strikingly, *VCAM1* and *ITGA4*, two of the top 10 upregulated genes functionally interact, suggesting upregulation of a whole pathway. This pathway is one of the most prominently affected pathways in this analysis. VCAM1 is a ligand of the heterodimeric receptor ITGA4/B1, whose subunits *ITGA4* and *ITGB1* are upregulated 7.31 fold and 1.5 fold, respectively. Moreover, the other ligand of *ITGA4/B1*, the ECM component *FN1*, is also upregulated 3.65 fold (Figure 4C). FN1 can also bind to the Integrin α4β7 receptor (White and Muro, 2011), however *ITGB7* is not upregulated in our dataset, arguing for *ITGA4/B1* as the relevant heterodimeric receptor. Ligand mediated local activation of the α4β1 Integrin heterodimeric receptor promotes directional migration of different cell types (Goldfinger et al., 2003; Nishiya et al., 2005) and therefore represented a promising candidate for inducing the vascular phenotypes caused by loss of TDP-43.

Next, we tested whether the increased mRNA expression of *ITGA4*, *ITGB1*, *FN1*, and *VCAM1* leads to increased protein expression. Protein expression of ITGA4 and FN1 is strongly increased upon TDP-43 KD compared to controls (Figure 4D and Supplementary Figure S4A), while ITGB1 expression is moderately increased. Unfortunately, we could not detect endogenous VCAM1 expression by immunoblot. Thus, TDP-43 is required to attenuate expression of FN1 and VCAM1 and their receptor ITGA4B1 in HUVECs.

TDP-43 is known to bind to RNA with the ability to regulate RNA stability and splicing (Polymenidou et al., 2011; Tollervey et al., 2011). However, we did not identify altered splicing of *FN1, ITGA4B1*or *VCAM1* RNA in HUVEC upon TDP-43 KD (data not shown). We next asked if TDP-43 protein binds to *FN1, ITGA4B1*or *VCAM1* RNA in HUVEC and thereby increases their expression upon TDP-43 KD. Consistent with previous reports we identified TDP-43 binding in coding and non-coding RNAs, introns, exons and UTRs (Supplementary Figure S6) by individual-nucleotide resolution cross-linking immunoprecipitation-high-throughput sequencing (iCLIP) with a binding preference for the sequence motif GTGTG (Supplementary Figure S6B). Despite *FN1* being among the top 30 bound transcripts (Supplementary Table S3) and binding of TDP-43 at the exon/intron boundaries of FN1 (Supplementary Figure S6C), TDP-43 KD does not alter its splicing. Prominent binding of TDP-43 to the 3′and 5′UTR of *FN1* (Supplementary Figure S6C) suggests regulation of transcript stability or translation (Glisovic et al., 2008; Costessi et al., 2014).

### *fn1b* is increased in *tardbp−/−; tardbpl−/−* mutants

**FIGURE 5:**
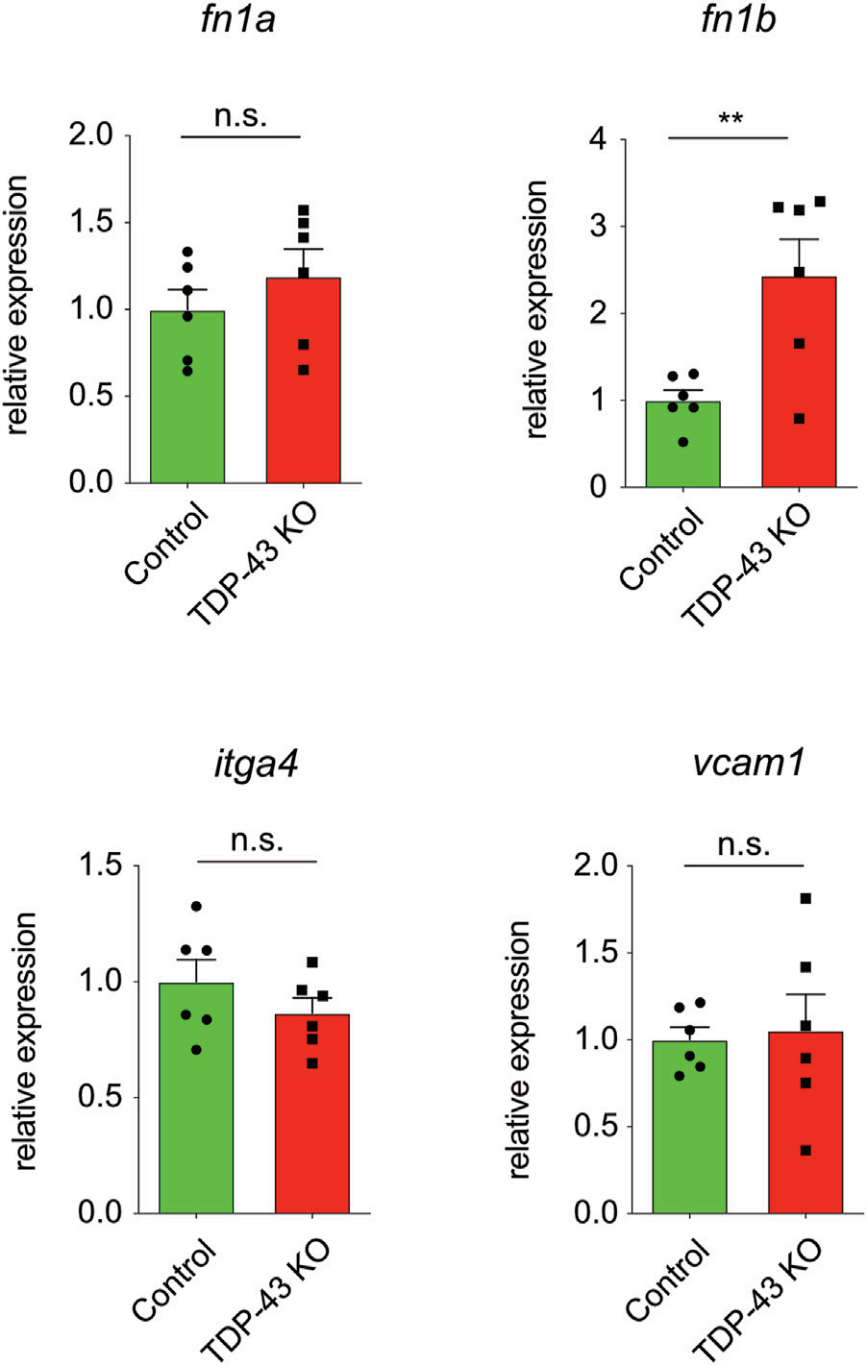
*fn1b* is increased in zebrafish. Relative mRNA expression of *fn1b*, but not *fn1a*, *itgα4*, or vcam1 is increased in *tardbp−/−; tardbpl−/−* mutants compared to their wild type siblings. n = 6 pools of embryos of independent clutches at 24 hpf, unpaired *t*-test. Results by qPCR were reproduced twice using the same cDNA.

We next analyzed whether the increase in expression of *ITGA4*, *FN1*, and *VCAM1* identified in HUVEC is also present in *tardbp−/−; tardbpl−/−* mutant zebrafish. *itgb1* levels were not analyzed, because first, zebrafish express six different paralogues of the human *ITGB1* and second, it was only mildly upregulated in HUVEC. Since zebrafish have two FN1 orthologues, *fn1a* and *fn1b*, mRNA expression of both were analyzed. *fn1b* mRNA levels are increased in *tardbp−/−; tardbpl−/−* whole animal samples compared to their wild type siblings, whereas *fn1a*, *itgα4* and *vcam1* levels are not (Figure 5). Potentially, increased levels of *fn1a*, *itgα4* and *vcam1* in the EC could be masked by unaltered levels in more abundant cell types.

### Lowering of *fn1b*, *itgα4*, and *vcam1* levels rescues hypersprouting of SA in *tardbp−/−; tardbpl−/−* mutants

**FIGURE 6:**
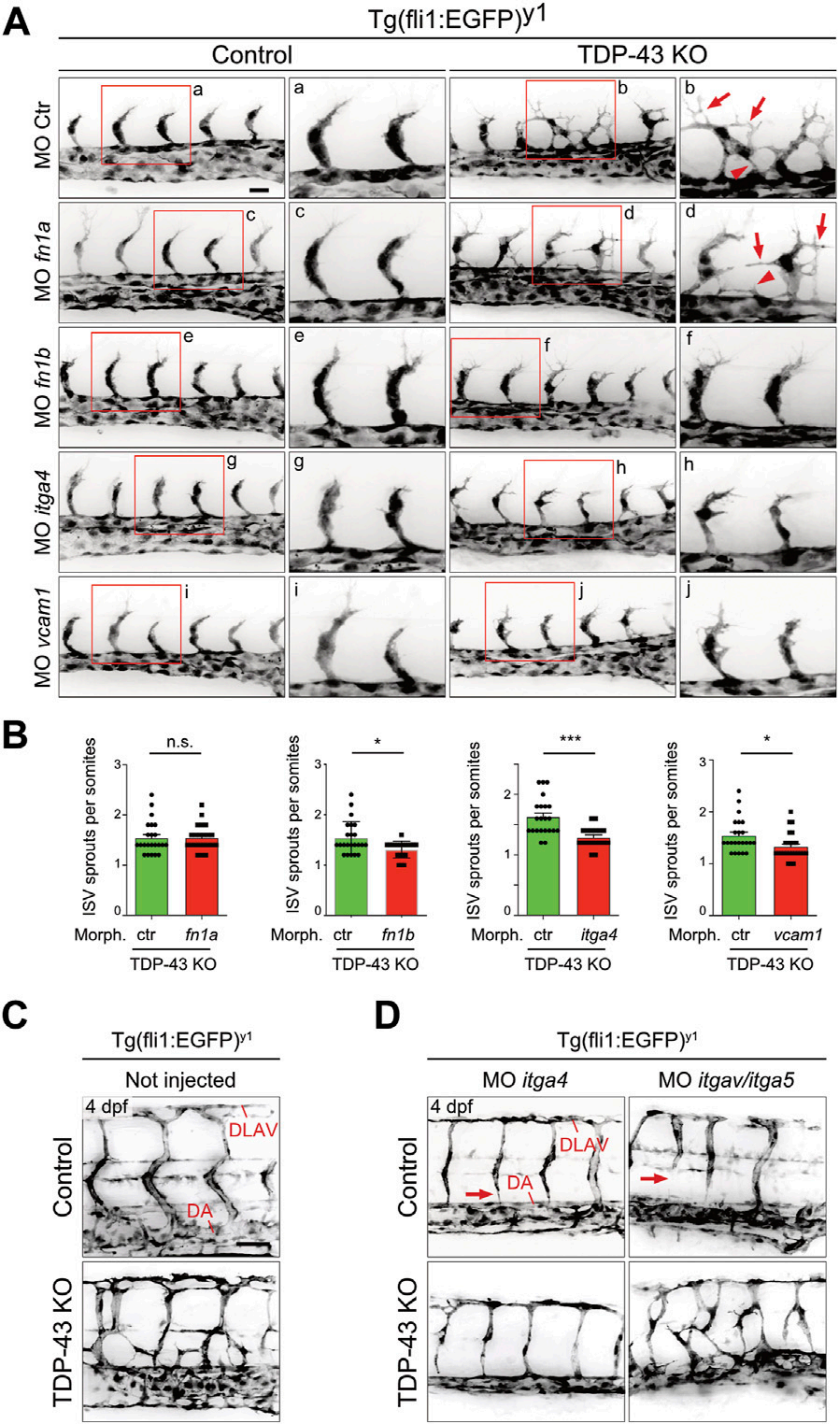
Mild KD of *fn1b*, *itgα4*, or *vcam1*, but not *fn1a* rescues increased SA sprouting in *tardbp−/−; tardbpl−/−* mutants. **(A)** Mild KD of *fn1a, fn1b*, *itgα4*, or *vcam1* does not affect SA growth in wild type embryos compared to ctr MO injected siblings. Images showing rescue results with normal SA sprout number in *tardbp−/−; tardbpl−/−* mutants injected with 0.33 mM *fn1b*, 0.5 mM *itgα4*, or 0.75 mM *vcam1* MO. Note the morphological difference compared to ctr MO injected *tardbp−/−; tardbpl−/−* mutants and the similarity to 0.75 mM ctr MO injected wild type sibglings. *fn1a* KD *tardbp−/−; tardbpl−/−* mutants (0.75 mM MO) are not rescued. **(B)** Statistical analysis demonstrating that mild KD of *fn1b*, *itgα4*, or *vcam1* can statistically significantly rescue increased sprouting of SA from the DA at 24 hpf in *tardbp−/−; tardbpl−/−* mutants compared to ctr MO injected *tardbp−/−; tardbpl−/−* mutants. One of three (*fn1a, fn1b, itgα4* KD) or two (*vcam1* KD) independent experiments with two clutches per experiment are shown, normality test and unpaired *t*-test, n ≥ 23. **(C)** Mutant tardbp−/−; tardbpl−/− and control embryos carry the Tg (fli1:EGFP)y1 transgene, SA of the trunk dorsal of the yolk sack extension are shown. **(D)** Representative image of 1 mM itgα4 MO and itgα5/itgαv MO injected sibling and tardbp−/−; tardbpl−/− mutant embryos (right panel). Note the normal number of SA of the itgα4 MO injected tardbp−/−; tardbpl−/− mutant. Arrows point to SA defects in wild type siblings injected with 0.5 mM itgα5 and 1 mM itgαv MO indicating lack of rescue activity. Scale bar 50 μm, anterior to the left.

If increased levels of *fn1b*, *itgα4* and *vcam1* in ECs is causative for the angiogenic defect in *tardbp−/−; tardbpl−/−* zebrafish embryos, then reducing their expression to approximately wild type levels should rescue the angiogenic phenotype. The developmental stage at which SA sprouts reach the horizontal myoseptum was chosen for quantification of angiogenic sprouting. To exclude off-site target effects of MO in general and to reduce impact of lowered expression of targeted genes in other tissues than the vasculature, low MO concentrations were chosen that do not impact SA growth in control MO injected embryos at 24 hpf (Figure 6A). However, as evaluated by the desired alterations in splicing patterns, successful partial KD of *itgα4* and *vcam1* is still detectable on RNA level (Supplementary Figure S7). Importantly, partial KD of *fn1b* but also *itgα4*, or *vcam1* rescued the hypersprouting phenotype of (Figures 6A, B). In contrast, KD of *fn1a* did not rescue this phenotype, consistent with the lack of upregulation in TDP-43 mutants (Figure 5, Figures 6A, B). The ability to rescue the mutant phenotype demonstrates that *fn1b*, *itgα4*, and *vcam1* upregulation is causative for the vascular phenotype in *tardbp−/−; tardbpl−/−* zebrafish.

The FN1 receptor Integrin α5β1 was previously implicated in angiogenesis *in vivo* (Astrof and Hynes, 2009). ITGAV is known to compensate the FN fibril assembly defect of ITGA5 knockout embryonic cells (Yang and Hynes, 1996). To circumvent a compensation of *itgα5* KD by *itgαv*, *itgα5* and *itgαv* MO were coinjected together and tested for rescue ability. Whereas combined KD of *itgα5* and *itgαv* did not rescue the vascular mis-patterning in any *tardbp−/−; tardbpl−/−* mutant, *itgα4* MO injection into *tardbp−/−; tardbpl−/−* mutants could strongly ameliorate the vascular phenotype (Figures 6A–C). *tardbp−/−; tardbpl−/−* embryos with *itgα4* KD have SA with nearly wild type morphology dorsal of the yolk sack extension. These findings underscore that the relevant integrin receptor α subunit of Fn1b causing the angiogenic defect of *tardbp−/−; tardbpl−/−* mutants is Itgα4.

## Discussion

One of the major problems identifying the physiological functions of TDP-43 *in vivo* has been the early embryonic lethality of the KO mouse (Kraemer et al., 2010; Sephton et al., 2010; Wu et al., 2010) and the large number of RNA targets it can bind and modulate (Polymenidou et al., 2011; Tollervey et al., 2011). We identified an essential function of TDP-43 in zebrafish and human cell culture in EC patterning. In zebrafish, KO of both TDP-43 orthologues is also embryonic lethal, however, due to the extra-corporal embryonic development of fish we uncovered an early embryonic severe EC phenotype (Schmid et al., 2013). In the SA of TDP-43 mutant zebrafish, EC sprout and extend lamellipodia into the somite area instead of being restricted to the somite boundaries and fail to migrate along their normal paths. In human EC branching is similarly increased. In an unbiased transcriptomics analysis, we identified *FN1*, *VCAM1*, and *ITGα4/β1* as upregulated genes upon loss of TDP-43 in HUVEC. In a previous study, *VCAM1* was also found to be 1.7 fold upregulated in adult mouse brain upon TDP-43 depletion (Polymenidou et al., 2011) supporting our findings. Additionally, in TDP-43 KO embryonic stem cells *FN1* was among other focal adhesion KEGG pathway members strongly mis-regulated (Chiang et al., 2010). In zebrafish we found *fn1b* RNA to be 2 fold upregulated, however the levels of *vcam1*, and *itgα4/β1* were not altered in whole embryo extracts. The zebrafish RNA sequencing was performed on whole embryos and gene expression changes in EC might be masked by lack of expression changes in other abundant cell types. Alternatively, basal EC expression of *itgα4* and *vcam1* might be very low keeping a potential increase in expression still below the detection limit. However, the rescue of the angiogenic defect with not only *fn1b,* but also *vcam1* and *itgα4/β1* MO supports the involvement of elevated *vcam1* and *itgα4/β*1 also in zebrafish vascular mis-patterning. Importantly, FN1/VCAM1/ITGα4/β1 signaling can induce the vascular mis-patterning distinct from the known key players in angiogenesis: the VEGFR2-pathway, NOTCH, ITGA5, and ITGAV. How loss of TDP-43 leads to *fn1b, vcam1,* and *itgα4/β*1 upregulation remains unclear. Interestingly, FN1 mRNA is a direct target of TDP-43 in HUVEC. We hypothesize that TDP-43 binding destabilizes the *FN1* mRNA since *FN1* levels are elevated upon loss of TDP-43. *VCAM1* and *ITGA4/Β1* levels most likely are regulated indirectly potentially by a feedback regulatory loop through increase of FN1 or by changes in microRNA expression.

Our data shows that Fn1 and Vcam1 binding to and activation of ItgA4/Β1 induces defects in directed migration of EC upon TDP-43 loss. Activation of ITGA4 has been previously shown to be required for directional migration of several cell types *in vivo* (Kil et al., 1998; Sengbusch et al., 2002; Grazioli et al., 2006; Lim et al., 2008) consistent with the zebrafish phenotype. Moreover, activation of ITGA4/Β1 induces and maintains polarity during directed migration (Goldfinger et al., 2003; Nishiya et al., 2005). Additionally, a prominent instructive role for directed cell migration of Fn has previously been demonstrated in myocardial precursors in zebrafish (Trinh and Stainier, 2004; Jessen, 2015) and directed migration of EC in mouse retinal angiogenesis (Stenzel et al., 2011) in line with our findings.

TDP-43 is the major pathological hallmark of ALS and our findings of its physiological function in angiogenesis is of potential disease relevance. Upon aggregation in the cytoplasm in disease state, TDP-43 is cleared from its normal nuclear localization and mis-regulated pathways through loss of TDP-43 contribute to disease. Several features of TDP-43 loss-of-function, such as splice alterations of *STATHMIN2* (Melamed et al., 2019; Prudencio et al., 2020), *UNC13A* (Brown et al., 2022; Ma et al., 2022) and cryptic proteins have been isolated from the ALS patient’s cerebral spinal fluid (Irwin et al., 2023; Seddighi et al., 2023) underscoring the clinical relevance of loss of TDP-43 consequences for ALS.

Misregulation of FN1 in motor neurons from sporadic ALS patients has been previously reported and might contribute directly to toxicity by altering the ECM-interaction of motor neurons and thereby affecting their vulnerability (Rabin et al., 2010; Prudencio et al., 2015). Alternatively, a TDP-43 driven vasculopathy could indirectly lead to motor neuron death in ALS. Evidence from TDP-43 inclusions in patient’s brain endothelial cells (Ferrer et al., 2021) and blood brain—and blood spinal cord barrier leakage in a conditional TDP-43 knockout mouse (Sasaki, 2015) support a direct TDP-43 dependent effect of vascular alterations to ALS pathology. Interestingly, a number of pro-inflammatory cytokines (*CXCL6*, *CXCL10*, *CXCL12*) have been identified to be highly upregulated by RNA sequencing in HUVEC upon TDP-43 KD (Supplementary Table S1), which might also contribute to disease progression in ALS by modulating the inflammatory response.

In summary, our study identified FN1/VCAM1-ITGA4B1 as a novel, important and evolutionary conserved molecular pathway regulated by TDP-43 with a potential mechanistic link to the pathophysiology of ALS.

## Data Availability

The original contributions presented in the study are publicly available. This data can be found as follows: The analysis as presented in Supplementary Figure S6 was processed by the iCount software (https://github.com/tomazc/iCount). Original iCLIP data is deposited at: https://imaps.goodwright.com/collections/993546811444/, https://imaps.goodwright.com/collections/285935488641/. The RNA sequencing data is deposited at https://www.ncbi.nlm.nih.gov/geo/query/acc.cgi?acc=GSE233588.

## Supplementary material

SUPPLEMENTARY FIGURE S1 Tardbpl Morpholino KD in *tardbp −/−* mutants fully phenocopies the tardbp/tardbpl KO. **(A)** Western blot indicating successful KD of Tardbpl_tv1protein levels in the wild type and in *tardbp −/−* mutant background in single embryos (note the high levels of Tardbpl_tv1 in tardbp -/- mutants in lane c compared to wild type in lane a compensating for the loss of Tardbp). Lane e are double homozygous *tardbp −/−* ; *tardbp −/−* mutants and in lane f are the corresponding pooled siblings with the genotype *tardbp +/- and tardbp +/+; tardbpl −/−* loaded. Tubulin serves as a loading control. **(B)** Tg(fli1a:EGFP)y1 highlights the vasculature shown in black. KD of Tardbpl in the tardbp-/- is phenotypically indistinguishable from the tardbp-/-; tardbpl-/- mutants (compare d with e). Scale bar 50 μm, anterior to the left. **(C)** There is no statistically significant difference between tardbp-/-; tardbpl-/- and tardbp-/- Tardbpl KD embryos in the average SA sprout number per somite and the ectopic sprout number per somite. Kruskal-Wallis test and Dunn’s multiple comparison post test, n ≥ 9, sprouts between boundaries of six somites dorsal to the end of the yolk sack extension were quantified.

SUPPLEMENTARY FIGURE S2 Arterial-venous differentiation and Notch target gene expression patterns are unaffected by TDP-43 KD. WISH with antisense probes specific for the Notch target and the arterial-venous differentiation markers ephrin-B2a and flt4 in tardbp-/- ctr MO injected embryos (Control) and their tardbp-/-; Tardbpl KD siblings at 24 hpf (Magnification: ×10 anterior to the left). Depicted are representative images of embryos from one clutch. Experiment was performed with embryos of three independent clutches.

SUPPLEMENTARY FIGURE S3 TDP-43 KD in HUVEC does not increase proliferation and shows no activation of the VEGFR2 signaling pathway. **(A)** Protein content as a measure of cellular survival is statistically significantly decreased upon TDP-43 KD with two TDP-43 targeting shRNAs. One-way ANOVA, n = 30 in three independent experiments. Cell death due to apoptosis and necrosis determined by quantification of LDH release is statistically significantly increased upon TDP-43 KD with two TDP-43 targeting shRNAs. One-way ANOVA, n = 30 in three independent experiments. KD of TDP-43 in HUVEC does not alter proliferation as shown by quantification of percentage of Ki67 positive cells. Unpaired t test, n = 9 in three independent experiments, *p*-value 0,5519. **(B)** Expression as well as phosphorylation levels of VEGFR2 signaling pathway components upon VEGF stimulation is not altered upon TDP-43 KD in HUVEC. Immunoblot of TDP-43 demonstrates successful KD upon transduction with shTARDBP#1 (KD) compared to shCtr transduced HUVEC (shCtr). VEGFR2, AKT, p38, PLCG, and ERK1/2 levels are not altered upon TDP-43 KD or upon stimulation with VEGF after 5/15/30 min. Also, phosphorylation levels of VEGFR2, AKT, p38, PLCG, and ERK1/2 are not altered upon TDP-43 KD or upon stimulation with VEGF after 5/15/30 min (pVEGFR2, pp38, pPLCG, pERK1/2). Time point 0 represents baseline levels after deprivation of growth factors before cells were stimulated with VEGF. Calnexin served as a loading control. Depicted is one representative experiment out of three independent experiments.

SUPPLEMENTARY FIGURE S4 Full Western blots. **(A)** Full Western blots for Fig. 4D. **(B)** Full Western blot for Fig. S3. Boxes contain corresponding membranes used as loading control and for quantification.

SUPPLEMENTARY FIGURE S5 Pathways analysis of NGS hits using Webgestalt.

SUPPLEMENTARY FIGURE S6 Individual-nucleotide resolution cross-linking immunoprecipitation (iCPLIP), sequence preference of TDP-43 binding in HUVEC and binding sites of TDP-43 to FN1. **(A)** Autoradiograph of 32P-labelled RNAs in the presence or absence of UV crosslinking and anti-TDP-43 antibody. High and low concentrations of RNase were used to confirm the presence of RNA bound to TDP-43. The asterisk marks the position in the gel corresponding to the size of a TDP-43 monomer. **(B)** Z-scores of pentamer occurrences surrounding (−30 nt to +30 nt) all TDP-43 cross-linked sites in HUVEC as determined by iCLIP. The sequences of the two most enriched pentamers are depicted. **(C)** Pie chart depicting the regional distribution of TDP-43 binding sites identified by TDP-43 iCLIP in HUVEC. TDP-43 cross-linked sites in the FN1 transcript as indicated by the TDP-43 iCLIP experiment in HUVEC cells (n = 2).

SUPPLEMENTARY FIGURE S7 itgα4 and vcam1KD strategy and MO validation. **(A)** Scheme illustrating the first three exons and two introns of zebrafish itgα4. Binding of the itgα4 MO (indicated in red) leads to skipping of exon2, as verified by sequence analysis of the resulting lower band at about 100 bp shown in **(B)**. Primers used for amplification are indicated by black arrows. B) PCR products of cDNA from ctr MO injected (-) or itgα4 MO injected (+) siblings of the embryos used in the rescue experiment shown in Figure 6. Injection of itgα4 MO leads to appearance of a second, lower migrating band representing itgα4 RNA lacking exon2. The upper band represents the normally spliced itgα4 RNA with exon1, 2, and 3. Bands were sequencing confirmed, revealing a frame shift resulting from exon skipping leading to a premature stop codon. **(C)** Scheme illustrating the first two exons and first intron of vcam1. Binding of the vcam1 MO (indicated in red) leads to partial inclusion of intron1 resulting in two RNAs with different length (see D). This was verified by sequence analysis. Primers used for amplification are indicated by black arrows. **(D)** PCR products of cDNA from ctr MO injected (-) or vcam1 MO injected (+) siblings of the embryos used in the rescue experiment shown in Figure 6. Injection of vcam1 MO leads to appearance of a second and third, higher migrating band representing vcam1 RNA with partial intron1 inclusion. The lowest band represents the normally spliced vcam1 RNA with exon1 and 2. Bands were sequence confirmed, revealing a frame shift resulting from partial exon inclusion leading to a premature stop codon.

## References

[B1] Astrof S. Hynes R. O. (2009). Fibronectins in vascular morphogenesis. Angiogenesis 12, 165–175. 10.1007/s10456-009-9136-6 19219555 PMC2716138

[B2] Brown A. L. Wilkins O. G. Keuss M. J. Hill S. E. Zanovello M. Lee W. C. (2022). TDP-43 loss and ALS-risk SNPs drive mis-splicing and depletion of UNC13A. Nature 603, 131–137. 10.1038/s41586-022-04436-3 35197628 PMC8891020

[B3] Bussmann J. Bakkers J. Schulte-Merker S. (2007). Early endocardial morphogenesis requires Scl/Tal1. PLoS Genet. 3, e140. 10.1371/journal.pgen.0030140 17722983 PMC1950955

[B4] Cannon J. E. Upton P. D. Smith J. C. Morrell N. W. (2010). Intersegmental vessel formation in zebrafish: Requirement for VEGF but not BMP signalling revealed by selective and non-selective BMP antagonists. Br. J. Pharmacol. 161, 140–149. 10.1111/j.1476-5381.2010.00871.x 20718746 PMC2962823

[B5] Carmeliet P. Tessier-Lavigne M. (2005). Common mechanisms of nerve and blood vessel wiring. Nature 436, 193–200. 10.1038/nature03875 16015319

[B6] Chi N. C. Shaw R. M. De Val S. Kang G. Jan L. Y. Black B. L. (2008). Foxn4 directly regulates tbx2b expression and atrioventricular canal formation. Genes Dev. 22, 734–739. 10.1101/gad.1629408 18347092 PMC2275426

[B7] Chiang P. M. Ling J. Jeong Y. H. Price D. L. Aja S. M. Wong P. C. (2010). Deletion of TDP-43 down-regulates Tbc1d1, a gene linked to obesity, and alters body fat metabolism. Proc. Natl. Acad. Sci. U. S. A. 107, 16320–16324. 10.1073/pnas.1002176107 20660762 PMC2941284

[B8] Childs S. Chen J. N. Garrity D. M. Fishman M. C. (2002). Patterning of angiogenesis in the zebrafish embryo. Development 129, 973–982. 10.1242/dev.129.4.973 11861480

[B9] Costessi L. Porro F. Iaconcig A. Muro A. F. (2014). TDP-43 regulates beta-adducin (Add2) transcript stability. RNA Biol. 11, 1280–1290. 10.1080/15476286.2014.996081 25602706 PMC4615836

[B10] Feiguin F. Godena V. K. Romano G. D'Ambrogio A. Klima R. Baralle F. E. (2009). Depletion of TDP-43 affects Drosophila motoneurons terminal synapsis and locomotive behavior. FEBS Lett. 583, 1586–1592. 10.1016/j.febslet.2009.04.019 19379745

[B11] Ferrer I. Andres-Benito P. Carmona M. Assialioui A. Povedano M. (2021). TDP-43 vasculopathy in the spinal cord in sporadic amyotrophic lateral sclerosis (sALS) and frontal cortex in sALS/FTLD-TDP. J. Neuropathol. Exp. Neurol. 80, 229–239. 10.1093/jnen/nlaa162 33421065 PMC7899266

[B12] Friedl P. Gilmour D. (2009). Collective cell migration in morphogenesis, regeneration and cancer. Nat. Rev. Mol. Cell Biol. 10, 445–457. 10.1038/nrm2720 19546857

[B13] Glisovic T. Bachorik J. L. Yong J. Dreyfuss G. (2008). RNA-binding proteins and post-transcriptional gene regulation. FEBS Lett. 582, 1977–1986. 10.1016/j.febslet.2008.03.004 18342629 PMC2858862

[B14] Goldfinger L. E. Han J. Kiosses W. B. Howe A. K. Ginsberg M. H. (2003). Spatial restriction of alpha4 integrin phosphorylation regulates lamellipodial stability and alpha4beta1-dependent cell migration. J. Cell Biol. 162, 731–741. 10.1083/jcb.200304031 12913113 PMC2173787

[B15] Grazioli A. Alves C. S. Konstantopoulos K. Yang J. T. (2006). Defective blood vessel development and pericyte/pvSMC distribution in alpha 4 integrin-deficient mouse embryos. Dev. Biol. 293, 165–177. 10.1016/j.ydbio.2006.01.026 16529735

[B16] Hao J. Ho J. N. Lewis J. A. Karim K. A. Daniels R. N. Gentry P. R. (2010). *In vivo* structure-activity relationship study of dorsomorphin analogues identifies selective VEGF and BMP inhibitors. ACS Chem. Biol. 5, 245–253. 10.1021/cb9002865 20020776 PMC2825290

[B17] Hewamadduma C. A. Grierson A. J. Ma T. P. Pan L. Moens C. B. Ingham P. W. (2013). Tardbpl splicing rescues motor neuron and axonal development in a mutant tardbp zebrafish. Hum. Mol. Genet. 22, 2376–2386. 10.1093/hmg/ddt082 23427147 PMC3658164

[B18] Hogan B. M. Schulte-Merker S. (2017). How to plumb a pisces: Understanding vascular development and disease using zebrafish embryos. Dev. Cell 42, 567–583. 10.1016/j.devcel.2017.08.015 28950100

[B19] Iguchi Y. Katsuno M. Niwa J.-I. Takagi S. Ishigaki S. Ikenaka K. (2013). Loss of TDP-43 causes age-dependent progressive motor neuron degeneration. Brain 136, 1371–1382. 10.1093/brain/awt029 23449777

[B20] Irwin K. E. Jasin P. Braunstein K. E. Sinha I. Bowden K. D. Moghekar A. (2023). A fluid biomarker reveals loss of TDP-43 splicing repression in pre-symptomatic ALS. bioRxiv. 10.1101/2023.01.23.525202 PMC10878965 38278991

[B21] Isogai S. Horiguchi M. Weinstein B. M. (2001). The vascular anatomy of the developing zebrafish: An atlas of embryonic and early larval development. Dev. Biol. 230, 278–301. 10.1006/dbio.2000.9995 11161578

[B22] Isogai S. Lawson N. D. Torrealday S. Horiguchi M. Weinstein B. M. (2003). Angiogenic network formation in the developing vertebrate trunk. Development 130, 5281–5290. 10.1242/dev.00733 12954720

[B23] Jessen J. R. (2015). Recent advances in the study of zebrafish extracellular matrix proteins. Dev. Biol. 401, 110–121. 10.1016/j.ydbio.2014.12.022 25553981

[B24] Julich D. Geisler R. Holley S. A. Tubingen Screen C. (2005). Integrinalpha5 and delta/notch signaling have complementary spatiotemporal requirements during zebrafish somitogenesis. Dev. Cell 8, 575–586. 10.1016/j.devcel.2005.01.016 15809039

[B25] Kemp H. A. Carmany-Rampey A. Moens C. (2009). Generating chimeric zebrafish embryos by transplantation. J. Vis. Exp., 1394. 10.3791/1394 19617875 PMC2770904

[B26] Kil S. H. Krull C. E. Cann G. Clegg D. Bronner-Fraser M. (1998). The alpha4 subunit of integrin is important for neural crest cell migration. Dev. Biol. 202, 29–42. 10.1006/dbio.1998.8985 9758701

[B27] Kimmel C. B. Ballard W. W. Kimmel S. R. Ullmann B. Schilling T. F. (1995). Stages of embryonic development of the zebrafish. Dev. Dyn. official Publ. Am. Assoc. Anatomists 203, 253–310. 10.1002/aja.1002030302 8589427

[B28] Kraemer B. C. Schuck T. Wheeler J. M. Robinson L. C. Trojanowski J. Q. Lee V. M. (2010). Loss of murine TDP-43 disrupts motor function and plays an essential role in embryogenesis. Acta Neuropathol. 119, 409–419. 10.1007/s00401-010-0659-0 20198480 PMC2880609

[B29] Lawson N. D. Scheer N. Pham V. N. Kim C. H. Chitnis A. B. Campos-Ortega J. A. (2001). Notch signaling is required for arterial-venous differentiation during embryonic vascular development. Development 128, 3675–3683. 10.1242/dev.128.19.3675 11585794

[B30] Lim C. J. Kain K. H. Tkachenko E. Goldfinger L. E. Gutierrez E. Allen M. D. (2008). Integrin-mediated protein kinase A activation at the leading edge of migrating cells. Mol. Biol. Cell 19, 4930–4941. 10.1091/mbc.e08-06-0564 18784251 PMC2575143

[B31] Liu J. Zeng L. Kennedy R. M. Gruenig N. M. Childs S. J. (2012). βPix plays a dual role in cerebral vascular stability and angiogenesis, and interacts with integrin αvβ8. Dev. Biol. 363, 95–105. 10.1016/j.ydbio.2011.12.022 22206757

[B32] Ma X. R. Prudencio M. Koike Y. Vatsavayai S. C. Kim G. Harbinski F. (2022). TDP-43 represses cryptic exon inclusion in the FTD-ALS gene UNC13A. Nature 603, 124–130. 10.1038/s41586-022-04424-7 35197626 PMC8891019

[B33] Melamed Z. Lopez-Erauskin J. Baughn M. W. Zhang O. Drenner K. Sun Y. (2019). Premature polyadenylation-mediated loss of stathmin-2 is a hallmark of TDP-43-dependent neurodegeneration. Nat. Neurosci. 22, 180–190. 10.1038/s41593-018-0293-z 30643298 PMC6348009

[B34] Montañez E. Casaroli-Marano R. P. Vilaró S. Pagan R. (2002). Comparative study of tube assembly in three-dimensional collagen matrix and on Matrigel coats. Angiogenesis 5, 167–172. 10.1023/a:1023837821062 12831057

[B35] Neumann M. Sampathu D. M. Kwong L. K. Truax A. C. Micsenyi M. C. Chou T. T. (2006). Ubiquitinated TDP-43 in frontotemporal lobar degeneration and amyotrophic lateral sclerosis. Science 314, 130–133. 10.1126/science.1134108 17023659

[B36] Nishiya N. Kiosses W. B. Han J. Ginsberg M. H. (2005). An alpha4 integrin-paxillin-Arf-GAP complex restricts Rac activation to the leading edge of migrating cells. Nat. Cell Biol. 7, 343–352. 10.1038/ncb1234 15793570

[B37] Petrie R. J. Doyle A. D. Yamada K. M. (2009). Random versus directionally persistent cell migration. Nat. Rev. Mol. Cell Biol. 10, 538–549. 10.1038/nrm2729 19603038 PMC2752299

[B38] Polymenidou M. Lagier-Tourenne C. Hutt K. R. Huelga S. C. Moran J. Liang T. Y. (2011). Long pre-mRNA depletion and RNA missplicing contribute to neuronal vulnerability from loss of TDP-43. Nat. Neurosci. 14, 459–468. 10.1038/nn.2779 21358643 PMC3094729

[B39] Prudencio M. Belzil V. V. Batra R. Ross C. A. Gendron T. F. Pregent L. J. (2015). Distinct brain transcriptome profiles in C9orf72-associated and sporadic ALS. Nat. Neurosci. 18, 1175–1182. 10.1038/nn.4065 26192745 PMC4830686

[B40] Prudencio M. Humphrey J. Pickles S. Brown A. L. Hill S. E. Kachergus J. M. (2020). Truncated stathmin-2 is a marker of TDP-43 pathology in frontotemporal dementia. J. Clin. Invest. 130, 6080–6092. 10.1172/JCI139741 32790644 PMC7598060

[B41] Quaegebeur A. Lange C. Carmeliet P. (2011). The neurovascular link in health and disease: Molecular mechanisms and therapeutic implications. Neuron 71, 406–424. 10.1016/j.neuron.2011.07.013 21835339

[B42] Rabin S. J. Kim J. M. Baughn M. Libby R. T. Kim Y. J. Fan Y. (2010). Sporadic ALS has compartment-specific aberrant exon splicing and altered cell-matrix adhesion biology. Hum. Mol. Genet. 19, 313–328. 10.1093/hmg/ddp498 19864493 PMC2796893

[B43] Roman B. L. Pham V. N. Lawson N. D. Kulik M. Childs S. Lekven A. C. (2002). Disruption of acvrl1 increases endothelial cell number in zebrafish cranial vessels. Development 129, 3009–3019. 10.1242/dev.129.12.3009 12050147

[B44] Sasaki S. (2015). Alterations of the blood-spinal cord barrier in sporadic amyotrophic lateral sclerosis. Neuropathology 35, 518–528. 10.1111/neup.12221 26242689

[B45] Schmid B. Hruscha A. Hogl S. Banzhaf-Strathmann J. Strecker K. Van der Zee J. (2013). Loss of ALS-associated TDP-43 in zebrafish causes muscle degeneration, vascular dysfunction, and reduced motor neuron axon outgrowth. Proc. Natl. Acad. Sci. U. S. A. 110, 4986–4991. 10.1073/pnas.1218311110 23457265 PMC3612625

[B46] Seddighi S. Qi Y. A. Brown A.-L. Wilkins O. G. Bereda C. Belair C. (2023). Mis-spliced transcripts generate *de novo* proteins in TDP-43-related ALS/FTD. bioRxiv. 10.1101/2023.01.23.525149 PMC11325748 38277467

[B47] Sengbusch J. K. He W. Pinco K. A. Yang J. T. (2002). Dual functions of [alpha]4[beta]1 integrin in epicardial development: Initial migration and long-term attachment. J. Cell Biol. 157, 873–882. 10.1083/jcb.200203075 12021259 PMC2173412

[B48] Sephton C. F. Good S. K. Atkin S. Dewey C. M. Mayer P., 3R. D. Herz J. (2010). TDP-43 is a developmentally regulated protein essential for early embryonic development. J. Biol. Chem. 285, 6826–6834. 10.1074/jbc.M109.061846 20040602 PMC2825476

[B49] Siekmann A. F. Lawson N. D. (2007). Notch signalling limits angiogenic cell behaviour in developing zebrafish arteries. Nature 445, 781–784. 10.1038/nature05577 17259972

[B50] Simons M. Gordon E. Claesson-Welsh L. (2016). Mechanisms and regulation of endothelial VEGF receptor signalling. Nat. Rev. Mol. Cell Biol. 17, 611–625. 10.1038/nrm.2016.87 27461391

[B51] Stenzel D. Lundkvist A. Sauvaget D. Busse M. Graupera M. Van der Flier A. (2011). Integrin-dependent and -independent functions of astrocytic fibronectin in retinal angiogenesis. Development 138, 4451–4463. 10.1242/dev.071381 21880786 PMC3177315

[B52] Tollervey J. R. Curk T. Rogelj B. Briese M. Cereda M. Kayikci M. (2011). Characterizing the RNA targets and position-dependent splicing regulation by TDP-43. Nat. Neurosci. 14, 452–458. 10.1038/nn.2778 21358640 PMC3108889

[B53] Torres-Vazquez J. Gitler A. D. Fraser S. D. Berk J. D. Van N. P. Fishman M. C. (2004). Semaphorin-plexin signaling guides patterning of the developing vasculature. Dev. Cell 7, 117–123. 10.1016/j.devcel.2004.06.008 15239959

[B54] Trinh L. A. Stainier D. Y. R. (2004). Fibronectin regulates epithelial organization during myocardial migration in zebrafish. Dev. Cell 6, 371–382. 10.1016/s1534-5807(04)00063-2 15030760

[B55] Van Rooijen E. Voest E. E. Logister I. Bussmann J. Korving J. Van Eeden F. J. (2010). von Hippel-Lindau tumor suppressor mutants faithfully model pathological hypoxia-driven angiogenesis and vascular retinopathies in zebrafish. Dis. Model Mech. 3, 343–353. 10.1242/dmm.004036 20335444

[B56] White E. S. Muro A. F. (2011). Fibronectin splice variants: Understanding their multiple roles in health and disease using engineered mouse models. IUBMB Life 63, 538–546. 10.1002/iub.493 21698758

[B57] Wu L. S. Cheng W. C. Hou S. C. Yan Y. T. Jiang S. T. Shen C. K. (2010). TDP-43, a neuro-pathosignature factor, is essential for early mouse embryogenesis. Genesis 48, 56–62. 10.1002/dvg.20584 20014337

[B58] Wu L.-S. Cheng W.-C. Shen C.-K. J. (2012). Targeted depletion of TDP-43 expression in the spinal cord motor neurons leads to the development of amyotrophic lateral sclerosis-like phenotypes in mice. J. Biol. Chem. 287, 27335–27344. 10.1074/jbc.M112.359000 22718760 PMC3431639

[B59] Xu Z. S. (2012). Does a loss of TDP-43 function cause neurodegeneration? Mol. Neurodegener. 7, 27. 10.1186/1750-1326-7-27 22697423 PMC3419078

[B60] Yang J. T. Hynes R. O. (1996). Fibronectin receptor functions in embryonic cells deficient in alpha 5 beta 1 integrin can be replaced by alpha V integrins. Mol. Biol. Cell 7, 1737–1748. 10.1091/mbc.7.11.1737 8930896 PMC276022

[B61] Yee C. S. Chandrasekhar A. Halloran M. C. Shoji W. Warren J. T. Kuwada J. Y. (1999). Molecular cloning, expression, and activity of zebrafish semaphorin Z1a. Brain Res. Bull. 48, 581–593. 10.1016/s0361-9230(99)00038-6 10386838

[B62] Zacchigna S. Lambrechts D. Carmeliet P. (2008). Neurovascular signalling defects in neurodegeneration. Nat. Rev. Neurosci. 9, 169–181. 10.1038/nrn2336 18253131

[B63] Zlokovic B. V. (2008). The blood-brain barrier in health and chronic neurodegenerative disorders. Neuron 57, 178–201. 10.1016/j.neuron.2008.01.003 18215617

[B64] Zygmunt T. Gay C. M. Blondelle J. Singh M. K. Flaherty K. M. Means P. C. (2011). Semaphorin-PlexinD1 signaling limits angiogenic potential via the VEGF decoy receptor sFlt1. Dev. Cell 21, 301–314. 10.1016/j.devcel.2011.06.033 21802375 PMC3156278

