# Supplementary figures and images for: Loss of TDP-43 causes ectopic endothelial sprouting and migration defects through increased fibronectin, vcam 1 and integrin α4/β1
Source: Front Cell Dev Biol. 2023 Jun 13;11:1169962. doi: 10.3389/fcell.2023.1169962 (PMC10299809; doi:10.3389/fcell.2023.1169962)

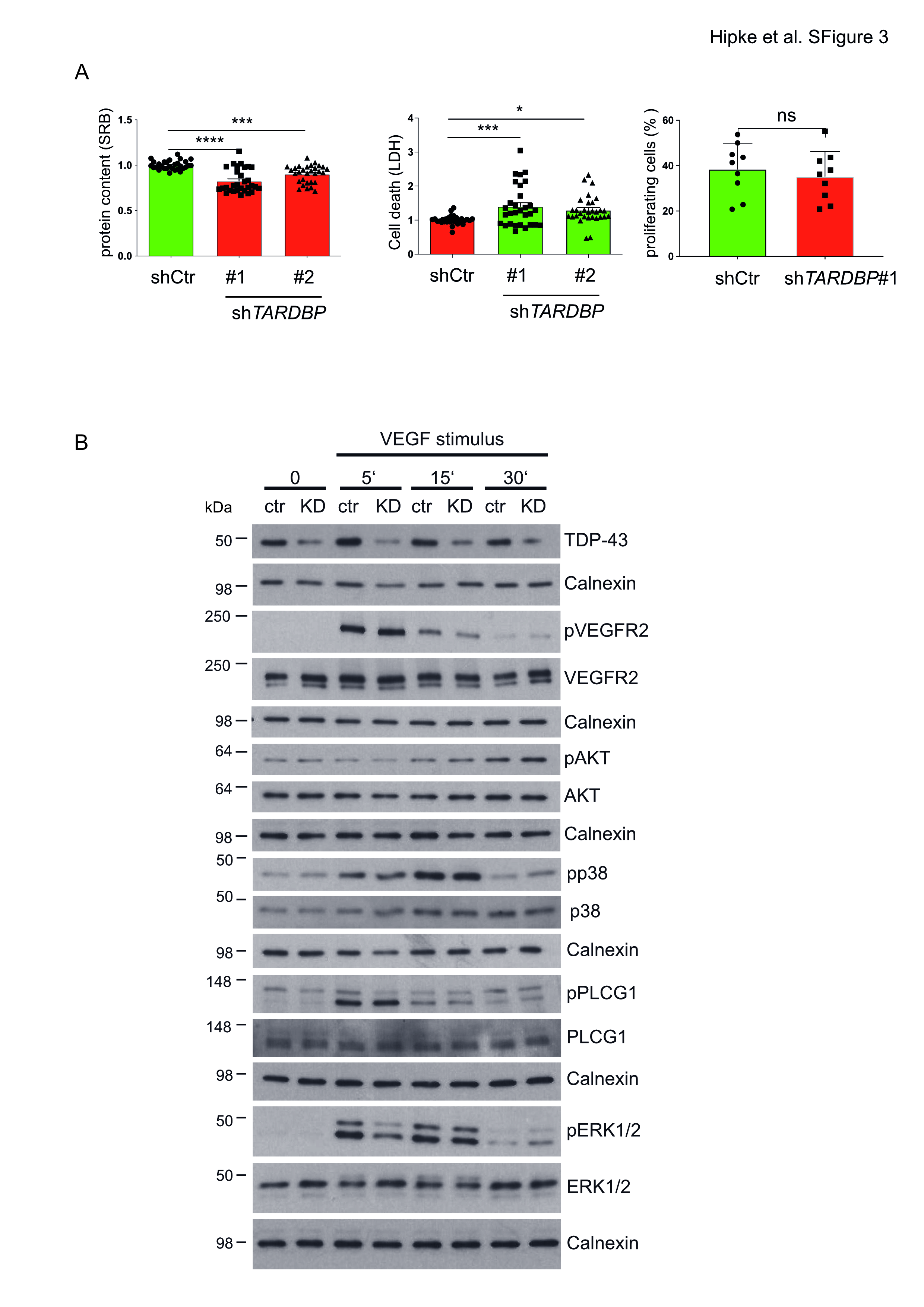

Supplement: Supplementary file 1 [file Image3.TIFF]

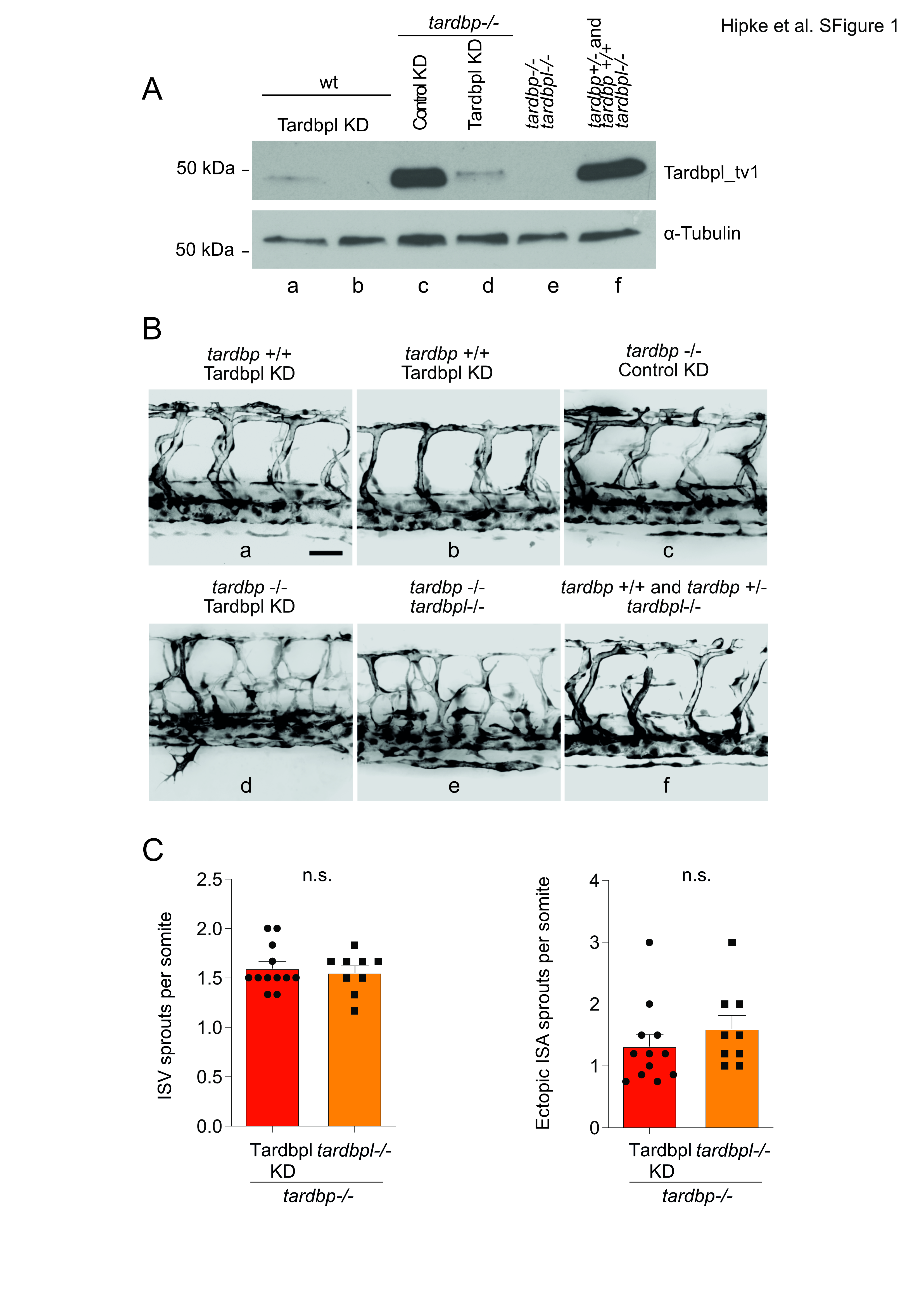

Supplement: Supplementary file 3 [file Image1.TIFF]

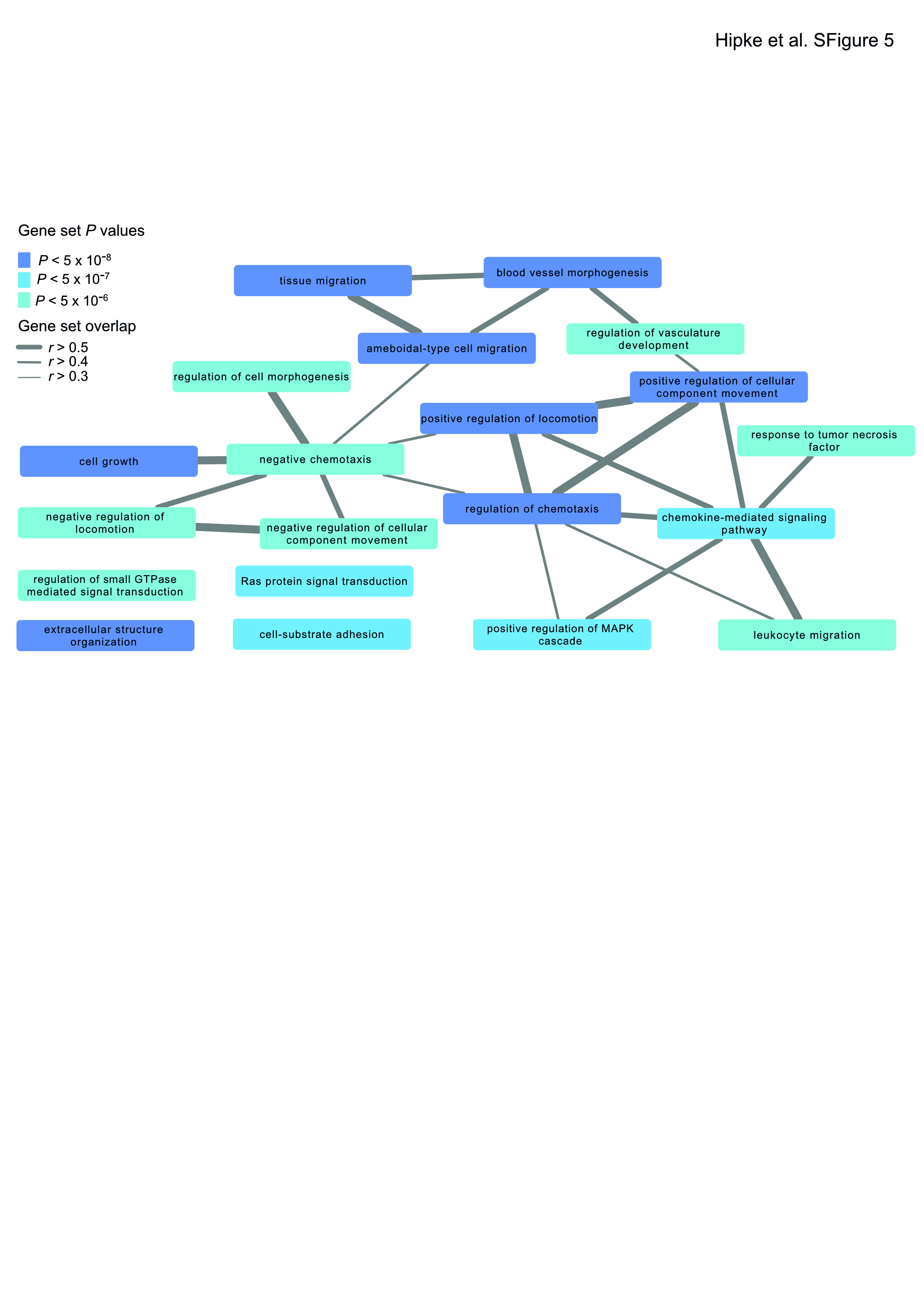

Supplement: Supplementary file 5 [file Image5.TIFF]

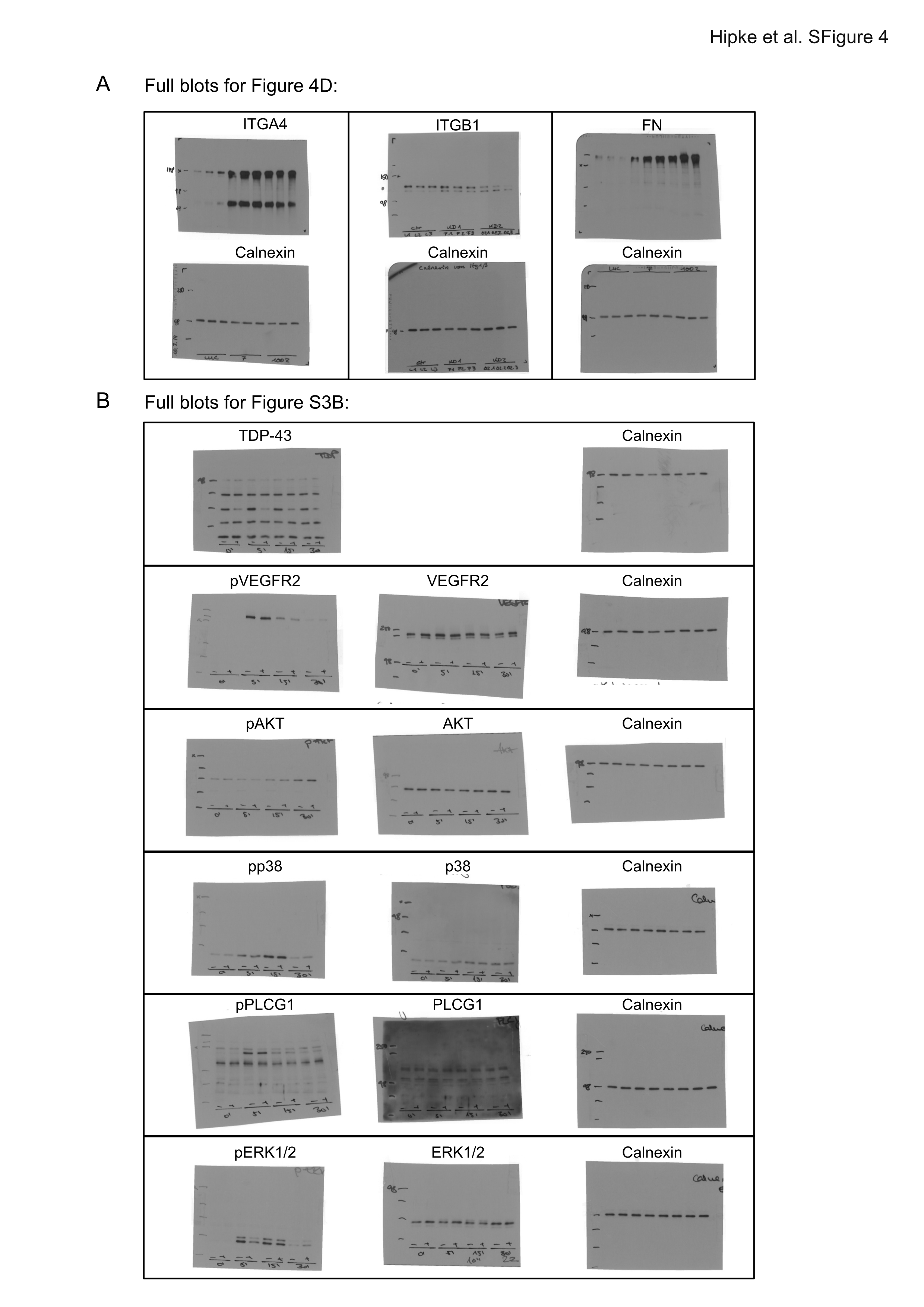

Supplement: Supplementary file 9 [file Image4.TIFF]

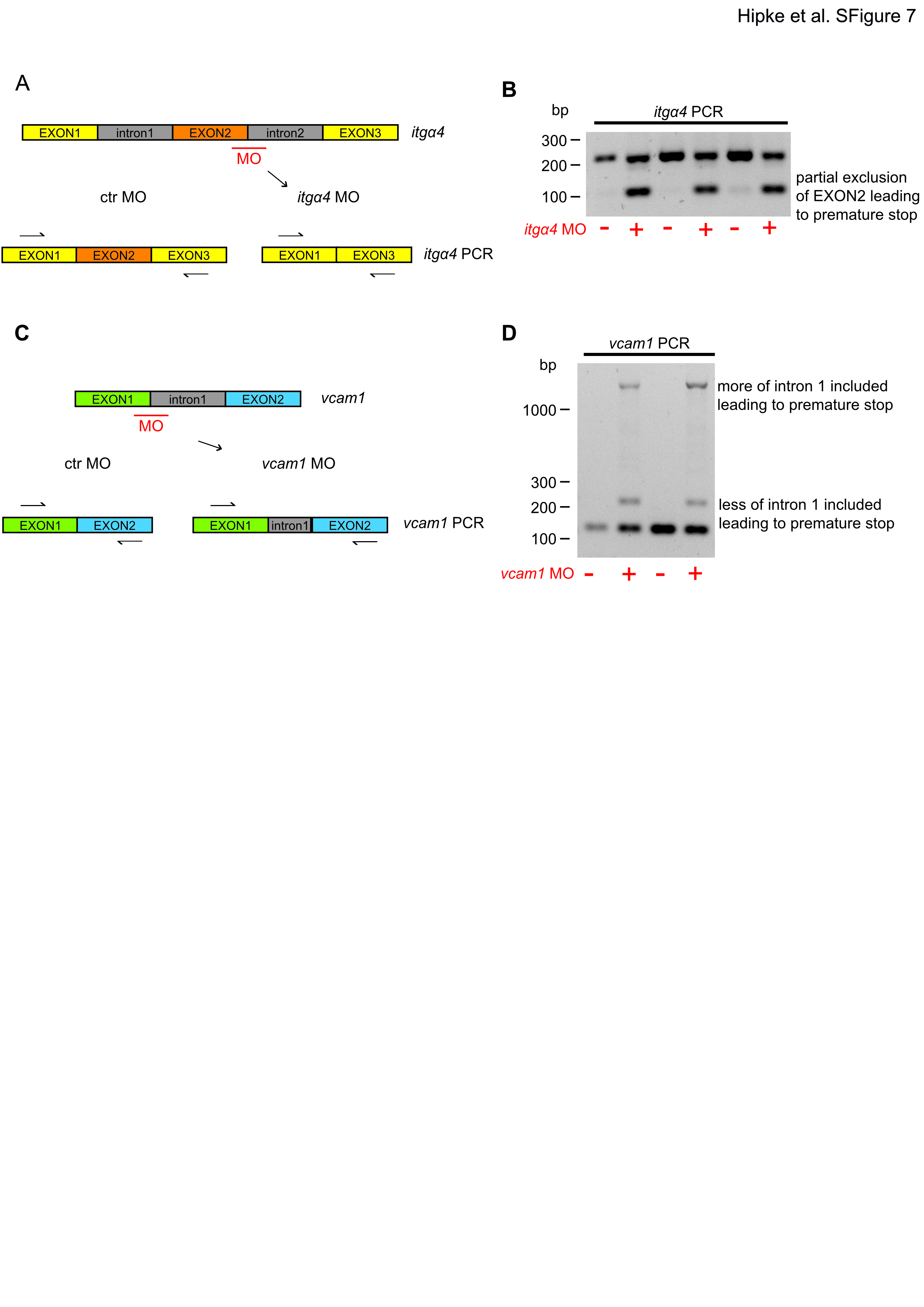

Supplement: Supplementary file 10 [file Image7.TIFF]
